# PROTOCOL: Interventions to increase youth employment: An evidence and gap map

**DOI:** 10.1002/cl2.1196

**Published:** 2021-10-18

**Authors:** Robert Apunyo, Howard White, Caroline Otike, Thomas Katairo, Sussana Puerto, Drew Gardiner, Alison A. Kinengyere, John Eyers, Ashrita Saran, Ekwaro A. Obuku

**Affiliations:** ^1^ Africa Centre for Systematic Reviews and Knowledge Translation, Makerere University College of Health Sciences, Makerere University College of Health Sciences Kampala Uganda; ^2^ Campbell Collaboration New Delhi India; ^3^ International Labour Organization Geneva Switzerland; ^4^ Sir Albert Cook Medical Library, Makerere University College of Health Sciences Kampala Uganda; ^5^ International Initiative for Impact Evaluation London UK

## Abstract

The research question guiding the production of the youth employment evidence and gap map (EGM) is stated as follows: What is the nature and extent of the evidence base of impact evaluations and systematic reviews on youth employment programmes in the world? The primary objective of is to catalogue impact evaluations and systematic reviews on youth employment interventions to enhance discoverability of evidence by decision makers, development patterners and researchers, so as to promote evidence‐based decision making in programming and delivery of youth employment initiatives. This evidence gap map is also a primary input into the implementation of Mastercard Foundation's strategy titled “Africa Works: Mastercard Foundation Strategy 2018–2030”, which points out sharing of evidence‐based knowledge and innovation with stakeholders as a key strategy to be used (Mastercard Foundation). The time frame for the development of the youth EGM will run from the last quarter of 2019 to December 2020. The five secondary objectives are: (i) To construct a framework for the classification of youth employment effectiveness studies. The objective will be achieved through the development of an intervention and outcome framework using an engaged consultative process involving the review team, Mastercard Foundation and other stakeholders. (ii) To identify available evidence, and clusters of evidence, including its quality rating. This will involve activities such as identification of studies using a standardised study search strategy, screening and coding of studies in EPPI Reviewer 4, which is a web‐based software program for production of reviews. (iii) To create a map of youth employment effectiveness studies equipped with an appealing user‐friendly web‐based search content visualisation using interactive mapping software. To achieve this object, data coded in EPPI Reviewer 4 will be exported to another software (EPPI mapper) which is designed for generating EGMs. (iv) To produce a narrative report of the youth employment EGM. This will be achieved through analysis of data in EPPI Reviewer 4 and report writing. To disseminate the EGM to users to increase awareness to support evidence‐informed decision‐making across countries. We will achieve this objective by organising dissemination workshops, participating in conferences and hosting the evidence and gap on our websites.

## 1 BACKGROUND

Globally, the population of young people is estimated at 1.2 billion, which is 16% of the world's population (United Nations, 2018). Worldwide, approximately 13% of young men and 30% of young women were classified as not in education, employment or training (NEET) in 2018. Youth who are NEET are almost excluded from the labour market because they are not gaining any skills to prepare them for future employment. Moreover, in the long run, a high NEET rate undermines the growth of a national economy over a sustained period (International Labour Organization [ILO], 2019).

In 2017, the global rate of youth unemployment was estimated at 13%, though Sub‐Saharan Africa and, Latin America and the Caribbean had the most disturbing situations. While youth unemployment in Sub‐Saharan Africa was 11.7%, some countries experienced extraordinarily high rates of youth unemployment. For instance, in South Africa youth unemployment rate was about 57.4%, and the highest in the region. In Lesotho, Mozambique and Namibia youth unemployment rates were estimated at 38.5%, 42.7% and 45.5% respectively. Similarly, in Latin America and the Caribbean, Argentina, and Brazil registered highest youth unemployment rates of 24.7% and 30.5% respectively, pushing the regional average to 18.9%, in 2017 (United Nations, 2018).

Moreover, most of the world and particularly Africa is experiencing high growth in youth population. The situation is envisaged to increase the continent's labour force to 375 million by 2030. The implication is that by 2035, there will be more young people in Africa available for the labour market each year than in the rest of the world combined (Mastercard Foundation, 2019). At a macro level, some drivers to youth unemployment include huge increases in labour supply, low aggregate demand for labour and, a mismatch between economic growth path and skills requirements majorly as a result of training deficits (De Lannoy et al., 2018).

Employment and economic empowerment are essential components of a strong foundation for all youth regardless of their gender identity and disadvantaged status. So, having decent work is crucial for young people and their future but it also has multiplier effects on local communities and the world as a whole (United Nations, 2018). Decent work refers to a composition of the aspirations of people in their working lives. It involves opportunities for work that are productive and deliver fair income, security in the workplace and social protection for families, better prospects for personal development and social integration (ILO, 2019a).

Addressing youth unemployment requires investment in job creation initiatives for the ever‐increasing population and tackling the skills mismatch problem contributing to the low school‐to‐work transition situation. Clearly, efforts to stimulate youth employment require diversification of employment sector through investment in education, skills training, bolstering small and medium‐sized enterprises and, microcredit provision (United Nations, 2018).

The UNDP Sustainable Development Goals (SDG) 8 and SDG 10 seek to reduce youth unemployment and inequality of all forms respectively (United Nations, 2019a). The SDG 8 sets three targets for youth employment. Target 8.5, expects countries, by 2030 to achieve full and productive employment and decent work for all women and men, including for young people and persons with disabilities, and equal pay for work of equal value. Target 8.6, expects countries, by 2020 to substantially reduce the proportion of youth not in employment, education or training and, target 8.b, by 2020, develop and operationalise a global strategy for youth employment and implement the Global Jobs Pact of the ILO (United Nations, 2019b). The SDG 10, “Reduce Inequality within and among countries” in Target 10.2, expects countries by 2030, to achieve empowerment and promotion of social, economic and political inclusion of all people irrespective of age, sex disability, race, ethnicity and economic status (United Nations, 2019c).

Consequently, global, regional and country based initiatives have been put in place to deal with youth unemployment. For instance, the United Nations Youth Strategy has been developed with several priorities of which the third talks about prompting economic empowerment through decent work, by supporting young people's greater access to decent work and productive employment (United Nations, n.d.). Similarly, Mastercard Foundation through its “Young Africa Works Strategy” has set out an ambitious goal to enable 30 million youth in Africa to secure jobs by 2030 through the promotion of sharing evidence‐based knowledge and innovation with stakeholders; supporting use of technology to drive impact and scale and; empowering young women (Mastercard Foundation, 2019).

To respond to the above, there is an increased need to invest in making available evidence on youth employment interventions discoverable by decision makers, development partners, researchers and other stakeholders. Evidence and gap maps (EGMs) can contribute to achieving this by identifying areas in which there are good bodies of synthesised knowledge to inform policy, and those areas in which there is little or no evidence synthesis to guide commissioning of coordinated research programmes.

### 1.1 Scope of the youth employment EGM^1^

Geographically, the proposed map will be global in coverage considering all countries regardless of their level of development. That means all world geographical regions and the World Bank country classification by income will be covered. The geographical regions are: Sub‐Saharan Africa, Latin America and Caribbean, East Asia and Pacific, Middle East and North Africa, South Asia, Europe and Central Asia, and North America. The World Bank country classification by income includes: low‐income countries, lower‐middle income, upper‐middle income, and high‐income countries (World Bank, 2020).

By population, the map will cover all young women and men aged 15–35 years from all countries. The three broad intervention categories include: strengthening training and education systems, enhancing labour market and, transforming financial sector market. The outcomes fall into five categories: education and skills, entrepreneurship, employment, welfare and economic.

In terms of evidence, the map will include impact evaluations of interventions aimed at increasing youth employment and systematic reviews of such single studies, published or made available between January 2000 and December 2019.

### 1.2 Why it is important to develop the youth employment EGM?

EGMs guide policy makers, development partners and researchers to available evidence to inform programme design and implementation of development interventions. Decision‐makers and researchers often lack awareness about the extent of evidence base, so maps help in increasing the discoverability, and thus use of that evidence for evidence‐informed decision making in international development policy and practice. The immediate potential application of the youth employment EGM is the operationalization of the Mastercard Foundation's strategic intervention that aims to promote sharing of evidence‐based knowledge and innovation with stakeholders for effective implementation of the “Africa Works: Mastercard Foundation Strategy 2018–2030” (Mastercard Foundation, 2019). For researchers, the youth employment EGM will identify research gaps for new primary research and new synthesis. This can inform strategic, policy‐oriented approach in commissioning relevant and coordinated research programmes (White at al., 2020).

However, the two existing evidence gap maps on youth employment related interventions are inadequate in a number of ways. For instance, these maps have a narrow scope (geographical, study publication period and, area/interventions and outcomes), and methodological limitations. The maps are limited to low‐ and middle‐income countries and publication period of 1990–2015. Moreover, development interventions are often best appreciated and understood in a contemporary context (Mawn et al., 2017). Further, both maps do not include economic outcomes while one does not include welfare outcomes. In addition, at least one of the maps suffered methodological problems which are said to have led to some studies being missed. Never the less, the two existing maps provide a basis for the development of the proposed youth employment EGM, with a broader focus (geographical, study publication period and, area/interventions and outcomes). Methodological limitations will also be avoided by better planning for sufficient time and human resources.

#### 1.2.1 The objectives

The research question guiding the production of the youth employment EGM is stated as follows: What is the nature and extent of the evidence base of impact evaluations and systematic reviews on youth employment programmes in the world?

The primary objective of is to catalogue impact evaluations and systematic reviews on youth employment interventions to enhance discoverability of evidence by decision makers, development patterners and researchers, so as to promote evidence‐based decision making in programming and delivery of youth employment initiatives. This evidence gap map is also a primary input into the implementation of Mastercard Foundation's strategy titled “Africa Works: Mastercard Foundation Strategy 2018–2030”, which points out sharing of evidence‐based knowledge and innovation with stakeholders as a key strategy to be used (Mastercard Foundation, 2019). The time frame for the development of the youth EGM will run from the last quarter of 2019 to December 2020.

The five secondary objectives are:

(i) To construct a framework for the classification of youth employment effectiveness studies. The objective will be achieved through the development of an intervention and outcome framework using an engaged consultative process involving the review team, Mastercard Foundation and other stakeholders.
(ii) To identify available evidence, and clusters of evidence, including its quality rating. This will involve activities such as identification of studies using a standardised study search strategy, screening and coding of studies in EPPI Reviewer 4, which is a web‐based software program for production of reviews.
(iii) To create a map of youth employment effectiveness studies equipped with an appealing user‐friendly web‐based search content visualisation using interactive mapping software. To achieve this object, data coded in EPPI Reviewer 4 will be exported to another software (EPPI mapper) which is designed for generating EGMs.
(iv) To produce a narrative report of the youth employment EGM. This will be achieved through analysis of data in EPPI Reviewer 4 and report writing.
(v) To disseminate the EGM to users to increase awareness to support evidence‐informed decision‐making across countries. We will achieve this objective by organizing dissemination workshops, participating in conferences and hosting the evidence and gap on our websites.

### 1.3 Defining EGMs

Saran and White (2018), define an EGM as “a systematic [visual] presentation of the availability of relevant evidence for a particular policy domain. The evidence is identified by a search following a pre‐specified, published search protocol. The map may be accompanied by a descriptive report to summarize the evidence for stakeholders such as researchers, research commissioners, policy makers, and practitioners” (p. 11). Its important to note that EGMs summarise what evidence exists but not what the evidence says. For instance, an EGM catalogues studies in a particular policy domain in terms of outcomes and interventions but does not say the magnitude of outcomes reported by the studies.

EGMs are useful in many ways. First, they guide policy makers, development partners and researchers to relevant available evidence to inform the design and implementation of development interventions. Decision‐makers and researchers often lack awareness about the extent of evidence base, so maps help in increasing the discoverability, and thus use of that evidence for evidence‐informed decision making in international development policy and practice (White et al., 2020). Second, they create awareness among implementing agencies where relevant evidence for their interventions is lacking, so that they can act accordingly by collecting evidence for the intervention they are supporting. Finally, maps identify research gaps for new primary research and new synthesis. This can therefore inform strategic policy‐oriented approach in commissioning relevant and coordinated research programmes (White at al., 2020).

## 2 EXISTING EGMS ON YOUTH EMPLOYMENT INTERVENTIONS

To our knowledge, there are two evidence gap maps on youth employment related interventions and outcomes. The descriptions provided below for each of the evidence gap maps point out associated strengthens and limitations which are of scope and methodological nature.

The first evidence gap map is the “Youth and Transferable Skills evidence gap map” produced by (Rankin et al., 2015). The map included 98 studies and is accessible at https://gapmaps.3ieimpact.org/evidence-maps/youth-transferable-skills-evidence-gap-map. The map included studies published or made available between 1990 and 2015. The included studies were searched from January to February of 2015. The map is restricted to low‐ and middle‐income countries. In terms of youth employment as a development area, the map has a narrow focus, covering only transferable skills interventions and associated outcomes. For, instance economic outcomes are not covered, yet these set of outcomes like “cost effectiveness” have an important bearing on the implementation of programmes. The map also suffered methodological problems due to time constraints. Its mentioned that the use of a single specialist to supervise and compile the search work as well as reliance on one person to screen studies on titles and abstracts, could have led to some studies being missed (Rankin et al., 2015). The map has an accompanying published narrative report which provides detailed information on areas such as results and methodology, which is a strength. In addition, this map used an extensive study search strategy covering 34 websites and 4 research registries.

The second map is the “Youth employment evidence gap map”, by International Labour Organization (2018), which included 113 studies and is available at: https://gapmaps.3ieimpact.org/evidence-maps/youth-employment-evidence-gap-map. The map is restricted to low‐ and middle‐income countries and included studies published or made available between 1990 and 2014 which are contained in a systematic review by (Kluve et al., 2017), titled “Interventions to improve the labour market outcomes of youth: A systematic review of training, entrepreneurship promotion, employment services and subsidised employment interventions”. Having one source of studies could be a major weakness of this map. The map does not include economic and welfare outcomes. In addition, we failed to find a narrative report accompanying the EGM, an indication of its absence. Although, a narrative report is an optional product in the production of an EGM (Saran & White, 2018), the absence or lack of access to such a document denies users vital information.

The above maps provide a basis for the development of the proposed youth employment EGM, with a broader focus (geographical, study publication period and, area/interventions and outcomes).

## 3 METHODOLOGY

### 3.1 Development of the framework for the youth employment EGM

Development of the framework is considered the most important, and often the most difficult part of developing an evidence map (White et al., 2020). The framework provides the structure or layout of the EGM and a primary resource in the development of the search strategy, screening and coding tools. So, framework development is one of the first activities undertaken in the production of an EGM. Our framework will be developed in the last quarter of 2019.

A typical framework for an effectiveness EGM refers to the matrix of interventions (in rows) and outcomes (in columns), developed through review of existing maps on a related policy domain, policy literature and consultations with stakeholders (Rankin et al., 2015).

The development of the framework for the youth EGM will be achieved through a consultative process involving authors of the map, Mastercard Foundation and stakeholders in the youth employment area. The consultative approach is proposed to capture a wide range conceptual and contextual positions of Mastercard Foundation and stakeholders involved in youth employment programming and implementation. The steps to be followed are described below.

First, using a workshop approach in Uganda, the EGM authors will construct a draft framework by brainstorming and reviewing existing EGMs that included impact evaluations of interventions to improve youth labour market outcomes and systematic reviews of such single studies. Dr. Howard White who is an expert in development evaluation and Dr. Ekwaro A. Obuku, an expert in evidence synthesis, will lead this activity.

Second, the draft framework will be shared with Mastercard foundation to capture their input. Its important to note that Mastercard Foundation (funder) will be engaged all through the project life by ensuring that they review study tools (study screening tool, coding sheet and, a dictionary of outcomes and interventions), that will be developed by the EGM authors. A stakeholders' meeting involving government officials, civil society, development partners and academia is proposed in Kampala, Uganda to carry out consultations on the framework.

Third, the EGM authors led by Dr. Saran Ashrita, a methods expert will have a training workshop in Uganda which will incorporate Mastercard Foundation's feedback on the draft framework into the final framework. Additional activities that will be undertaken in this stage include drafting of the study screening tool, coding sheet, definitions of interventions and outcomes to guide coding of studies. Training people who will code studies using EPPI Reviewer 4, a web‐based software program for managing and analyzing data in literature reviews, will also be carried. The other activity will be piloting the framework with about 100 studies.

Finally, stakeholders' consultations will be conducted in the last quarter of 2019 with relevant officials in Uganda from government ministries, departments, agencies, private sector agencies, civil society organisations, vocational training institutes, international development agencies as well as academia. Specifically, the stakeholders include relevant officials from: Ministry of Gender, Labour and Social Development, Ministry of Public Service, Ministry of Finance Planning and Economic Development, Ministry of Local Government, Youth Members of Parliament, Enterprise Uganda, Private Sector Foundation Uganda, USAID Uganda Office, Uganda Youth Development Link, Makerere University Business School, Office of the Prime‐Minster, Office of the President and, Nakawa Vocational Training Institute. The meeting will be held in Uganda because a significant number of authors who will code studies are resident in Uganda, at the Africa Centre for Systematic Reviews and Knowledge Translation. Moreover, there are plans to disseminate the EGM in Uganda.

The objectives of the stakeholder consultations are to:

(i) Sensitize audience to the upcoming map
(ii) Consult audience on the proposed EGM framework
(iii) Present existing evidence on youth employment
(iv) Understand government policy priorities in this area, and evidence demands of other stakeholders.

### 3.2 Criteria for including and excluding studies

A review of 15 agencies by Saran and White (2018), established that the inclusion criteria for EGMs follows the PICOS approach of population, intervention, relevant comparison groups, outcomes and study design; though specifying comparisons is usually not included in map inclusion criteria. The inclusion criteria of the proposed youth employment evidence and gap will use the PICOS approach with the exception of “outcomes” and “comparison” components. In other words, studies will not be excluded basing outcomes (except where outcomes of youth are combined with those of ineligible populations), and comparison group aspects. Additional criterion of study publication period included.

**Figure 1:**
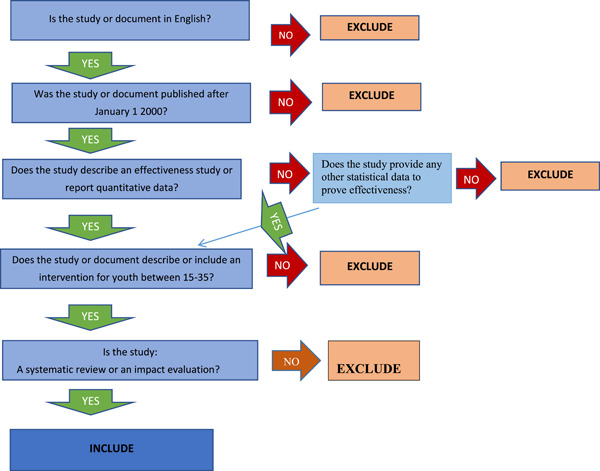
Screening tool

Therefore, the criteria for screening studies will include study design, publication or study availability period of between 2000 and 2019, description of an effectiveness study or study reports quantitative data and, intervention for youth aged 15–35 years (Figure 1). One study available in different versions may be included in the EGM multiple times if the versions employed different study designs or report different outcomes. However, for multiple papers/documents of a single study (i.e., working paper and journal article), only one with the most detailed information, including interventions and outcomes will be included in the EGM. For instance, where a journal article presents partial outcomes, a working paper will be included in the EGM.

For studies that combine youth and nonyouth (older adults and children), if outcomes for youth and other ineligible populations are reported separately, they will be included but excluded when outcomes are combined. In summary, generally studies will not be excluded on outcomes except where reported outcomes for youth and ineligible populations are combined. In addition, studies containing only a subset of eligible interventions shall be included in the map if revenant outcomes are reported.

### 3.3 Population

The only criterion for selecting the target population is age. So, the target population is all youth or young women and men aged 15–35 years from all countries. We acknowledge the diversity of varying national definitions of youth. For instance, while the United Nations defines youth as young women and men aged 15–24 years, in South Africa youth fall in the age group 14–35 years (South Africa, 2015), in Zimbabwe its from 15–35 years of age (Zimbabwe, Ministry of Youth Development, 2000), and in Uganda youth are within the age bracket of 15–30 years (Uganda, Ministry of Gender, Labour and Social Development, 2016). This EGM identifies youth as young women and men aged between 15 and 35 years.

Population subgroups include: both females and males; youth with disabilities; youth in fragility, conflict and violence (FCV) contexts; youth from disadvantaged background (low‐income families or low education); criminal background; ethnic minorities; and humanitarian settings. The population subgroups permit the identification of studies reporting evidence on equity.

### 3.4 Intervention(s) or the problem

The EGM will have three broad intervention categories, each with subcategories. The intervention categories that are proposed include: Strengthening training and education system; enhancing labour market; and transforming financial sector market. Table 1 lists the intervention categories, subcategories as well as examples to aid with study search and coding. However, when coding studies, any other interventions that may not fall within the above three categories but improve labour market outcomes of youth, shall be included in the map. Interventions whose aim is not about increasing youth employment will be excluded. Definitions of the interventions are provided in Supporting Information Annex 1, with a reference list provided as Supporting Information Annex 4.

**Table 1:** Intervention categories, subcategories and examples/descriptions

Category	Subcategory	Example
Strengthening training and education system	Training, up‐skilling and retraining/re‐skilling	Prior Learning Assessment and Recognition (PLAR)
		Education, technical and vocational training (TVET)
		Internship and apprenticeship
		Training centre accreditation and certification
		Training of trainers and teachers
		Business skills training
		Life skills training
Enhancing labour market	Support to employment	Employee Mentoring (Work integrated learning; on job training)
		Career offices/advisory services/career days
		Programme for overseas employment
		Public work programs
		Support to employee mobility and placements
		Wage subsidies
	Decent work policies	Labour standards
		Social protection and social security
		Accountability systems
	Information	Labour market information
		Digital services and SMS coaching
		Social media campaigns and awareness campaigns
		Value chain development
		Access to services and markets (value chains)
Transforming financial sector market	Entrepreneurship promotion and financing	Small and medium sized Enterprise finance (SME)
		Microfinance (to individuals)
		Social impact bonds
		Crowd funding
		Loan guarantees
		Grants
		Self‐financing groups
		Micro‐franchising

### 3.5 Outcomes

Table 2 contains proposed outcome categories and subcategories. The outcomes are arranged in a typology of five categories: education and skills, entrepreneurship, employment, welfare, and economic. We will flag up welfare outcomes to ensure that even welfare outcomes not directly associated with employment are captured. For instance, some welfare outcomes (criminal and delinquent behaviour as well as citizenship values) may result regardless of whether the youth (homeless youth) get employment after training. Adverse and unintended outcomes will be included in the map. This is important to avoid one‐sided summaries of the evidence. An example of unintended employment outcomes could be the youth offending such as, increased rate of alcohol abuse due to income resulting from change in employment status. An example of an adverse outcome is accidents and disease resulting from employment hazards. We will also code multiplier effects, which refer to effects not directly from the programme such as, youth spending earnings to improve local commerce and job displacement.

**Table 2:** Outcome categories and subcategories

Category	Subcategory
Economic	Costs
	Cost Benefit
	Cost effectiveness
	Multiplier, displacement and spillover effects
Education and skills	Education completion and qualifications
	Access to/in education
	Education quality
	Technical skills and vocational training
	Digital skills
	Transferable skills (including life and social skills, e.g., networking, negotiation)
Entrepreneurship	Access to financial services
	Business creation
	Business performance
	Job creation (Jobs for other people, e.g., number of employees)
Employment	Vacancies
	Actively seeking employment
	Employment expectation
	Employment status (including duration)
	Employment consistent with education/training
	Hours worked
	Job quality (include formal vs informal here)
	Earnings and salary
Welfare	Economic outcomes (except earnings). This also includes income at household level
	Criminal and delinquent behaviour (antisocial behaviour)
	Citizenship, values and social behaviour (Social behaviour is such things as taking part in community activities [clarifying to distinguish from antisocial behaviour]. Social behaviour: alcohol/drugs, hanging out with friends)
	Family health & education
	Inclusion and empowerment (social network). (Engagement in community activities is here [not social behaviour])

Definitions of outcomes are provided in Supporting Information Annex 2, with a reference list provided as Supporting Information Annex 4.

### 3.6 Summary of PICOS

 

**Table 3:** Summary of PICOS

Population	All youth or young women and men aged 15–35 years from all countries
Intervention	All interventions that fall under: strengthening training and education system; enhancing labour market and; transforming financial sector market
Comparison	Active or passive (placebo or non‐intervention) in the comparison group. This criterion will not be used to exclude studies
Outcomes	All outcomes categorised under economic, education and skills, entrepreneurship, employment, and welfare. This criterion will not be used to exclude studies, except where outcomes of youth are combined with those of ineligible population groups
Studies	The study must be both impact evaluations of youth employment interventions or systematic reviews, which included studies on youth employment interventions

### 3.7 Connection between interventions and potential outcomes

The constraints faced by youth with regard to participation in employment are widely documented nationally and globally. For instance, Datta et al. (2018), provides a typology of key constraints for youth employment such as:

- Gaps and mismatches in technical, cognitive and socioemotional skills that results from deficient education and training systems.
- Asymmetric information, whereby youth often lack information due to information gaps, little or no work experience and limited access to social networks.
- Lack of assets and limited access to credit; which excludes young people from engaging in productive self‐employment opportunities especially among rural youth and economies where agriculture is the most dominant productive activity.
- Regulatory constraints to hiring youth. Decent work policies can deter employers from hiring young new employees. For instance, employee protection legislation and mandatory social benefits may discourage hiring first‐time job seekers who may be higher risk.
- Limited access to credit and lack of assets. Young people usually have low savings, and limited assets for securing loans from formal financial institutions. These constraints exclude youth from financial inclusion and becoming entrepreneurs.

To address the above constraints, countries are designing and implementing various programmes. The youth employment interventions considered for this EGM are clustered in to three categories, which except education are described by Kluve et al. (2017) as active labour market programmes (ALMPs).^2^ The rationale for focusing on the three intervention categories namely: strengthening training and education systems, enhancing labour markets, and transforming financial sector markets stems from the generally broad scope of the youth employment EGM, attempting to include all youth employment interventions and associated outcomes. We provide the relationship between interventions and potential outcomes shown in Tables 1 and 2, respectively. Kluve et al. (2017) in Tables 1, 2, 3 document the “intervention—results chain” for different types of ALMPs, which are useful guides for tracing the intervention–outcome relationships.

First, “strengthening training and education systems” category of interventions equip youth with skills which are necessary for increasing employment opportunities in today's labour market (United Nations, 2018). Globally, skills training is the most widely implemented set of labour market interventions for youth (Kluve et al., 2017). Training programmes often equip youth with skills required by employers. Training and education interventions are also considered the most costly to implement in terms of financial and human resources. However, training and formal education interventions often do not reach marginalised youth especially young women, indigenous groups, or youth with disabilities, leaving them without the skills needed to realize their potential (United Nations, 2018). Skills attained through training can be categorised into technical skills, business skills and life or soft skills (Kluve et al., 2017). Technical skills are achieved by individuals attending training initiatives such as technical and vocational education and training (TVET) and, internship and apprenticeship. Business skills training is normally provided to increase entrepreneurial activities among youth (Kluve et al., 2017). In the case of life skills training, the objective is to strengthen trainees' self‐esteem and work habits to help them achieve the goals set by employers (Lippman et al., 2015). The “training and skills development interventions results chain”, diagnostic framework documented by Kluve et al. (2017) is a useful resource for mapping the relationship between training and education intervention and potential outcomes. Participation in training and education is expected to create mainly three categories of outcome: (i) employment outcomes (i.e., change in employment status, earning and salary); (ii) education and skills outcomes such as acquisition of technical and vocational skills; and (iii) economic outcomes such as cost benefit and cost effectiveness (Table 2).

Second, “enhancing labour market” category of interventions will be grouped into three subcategories including: support to employment; enhancing labour market; and transforming financial sector market (Table 1). Support to employment interventions are generally meant to help youth find jobs through provision of jobs via initiatives like programmes for overseas employment and public works programmes. Employment services also facilitate the youth in the process of finding jobs through career guidance and supporting employee mobility needs. Decent work polices generally regulate the relationship between employers and employees in the employment environment through application of labour standards, regulations and accountability systems. Another enhancer of labour markets, is provision of a wide range information about the labour market and associated services (Table 1). Potential outcomes from this category of interventions tend to be largely associated with employment outcomes such as job quality and change in employment status (Table 2).

Finally, “transforming financial sector” category of interventions focus on entrepreneurship promotion and financing. The category of interventions can be considered the most popular for targeting disadvantaged youth especially those excluded from training and education programmes. Datta et al. (2018) notes that labour market opportunities are significantly influenced by the reverence of skills for the existing job market. An intervention like self‐help or financing groups tend to be dominant among youth with less education and training. Self‐help groups are small groups that save a certain amount of money on weekly or monthly basis and issue loans to members out of their collective savings (Flynn, 2013). The category of interventions (transforming financial sector) tend to be mainly associated with welfare outcomes and employment outcomes (Table 2) than economic outcomes.

### 3.8 Study designs

The selected study designs described below are the appropriate designs for estimating effectiveness of program interventions. The designs (experimental and nonexperimental) are for conducting impact evaluations of development interventions. Examples of non‐evaluation‐based study designs that shall be excluded are ethnography, case control and cross‐sectional. For instance, the focus of a cross‐sectional study is limited to data from a particular population with variables of interest, at a given point in time. Cross‐sectional studies are observational in nature and not causal implying that they are not applicable for determining the effect of an intervention.

a) *Experimental designs*

Experimental designs fall into two types, namely randomised controlled trials (RCTs) and natural experiments.

(i) RCTs: A typical RCT design involves randomising study participants into two or groups (an experimental/treatment/intervention group and control group) in which the researcher introduces an intervention and measures its impact on the dependent variable at least two times namely pre‐ and posttest measurements (White & Sabarwal, 2014).
  One of the weaknesses of an RCT study design is that it suffers from missing outcomes resulting from changes that occur post randomisation of study participants. For instance, withdrawal of subjects from the study and noncompliance with established study protocols or guidelines would lead to missing outcomes (White & Sabarwal, 2014). Therefore, application of Intention‐to‐treat (ITT) analysis in RCTs attempts to address this problem by including in the analysis every subject who is randomised according to randomised treatment assignment and ignoring anything that happens after randomisation (Gupta, 2011). So, studies using ITT analysis will be coded under RCT study design.
(ii) Natural experiments: Despite the lack of universally accepted definition of the term natural experiment, researchers are in agreement that a natural experiment happens where and/or when an intervention is implemented without the control of a researcher (Butler et al., 2018). For example, a policy development emphasising promotion of TVET in Uganda can be seen as a natural experiment. TVET is a school‐based intervention, which makes it a commonplace intervention, and so a natural experiment could be politically and ethically a feasible evaluation study design.

b) *Nonexperimental matching designs*

Nonexperimental designs are used where random assignment is not feasible for various reasons. For instance, where the need for evaluation arises when the program is completed or on going. Nonexperimental designs can be generally categorised as quasi‐experimental and regression‐based approaches. Quasi‐experimental methods create comparison groups by statistical methods, rather than by random assignment. These methods include difference‐in‐differences (DiD), propensity score matching (PSM), regression discontinuity designs (RDD), and synthetic controls designs (White & Raitzer, 2017).

(i) PSM: In PSM, the matching enables construction of an artificial comparison group with almost similar characteristics as the treatment group. The artificially created comparison group from untreated observations is matched to treatment observations from the untreated sample, based on observable characteristics. The treated units are matched to untreated units with a similar propensity score. The matching approach is considered adequate to attain an unbiased impact estimate (White & Sabarwal, 2014).
(ii) DiD: In DiD approach, impact is estimated by comparing the changes in outcome over time between treatment and comparison groups. The method is also known as controlled before and after studies or “double difference” method (White & Sabarwal, 2014).
(iii) RDD: This is a popular approach used in econometrics due to situations that make randomisation unfeasible to determine causal effects of interventions by assigning a cutoff or threshold above or below which an intervention is assigned. The threshold refers to the criterion that participants must met before being included in the intervention. The threshold is usually based on a continuous variable (White & Sabarwal, 2014). For instance, adults above or below a particular age for enroling in a training programme. RDD approach compares observations on either side of the threshold to estimate average treatment effects of an intervention. The major limitation of RDD is that its greatly affected by confounding variables. For instance, average treatment effects of a local sanitation intervention may be affected by a regional related intervention if they were implemented at the same time.

c) *Regression‐based approaches*

All approaches based on regression models not listed above are included here. These include (but are not restricted to):

(i) Instrumental variable (IV): “A statistical technique for estimating causal relationships when an RCT is not feasible or when an intervention does not reach every participant/unit in an RCT” (White & Sabarwal, 2014, p. i).
(ii) Control function approaches (e.g., Heckmann model): A two‐step approach in which a participation equation is first estimated, which is used to generate fitted values which are used in the second stage equation estimating the outcome.
(iii) Endogenous switching regressions: The approach models two outcome equations (two “regimes”), one for treatment and one for comparison, allowing for endogeneity of selection into treatment.

d) *Systematic reviews*

A systematic review summarises bodies of literature with summary statements of the findings of that literature. The term “systematic” in systematic review refers to the research process that is intended to minimise the biases that may occur in a traditional literature review. Key characteristics of a systematic review include: a clear scope for the review; set of research questions; clear study inclusion and exclusion criteria; systematic search strategy used to identify the single studies that would meet the eligibility criteria; and results. Therefore, studies to be included in the EGM as systematic reviews shall have the above‐mentioned characteristics even if they do not use the term “systematic” in the titles. Studies using the term “systematic” but lack key features of a systematic review will be excluded. For instance, scoping reviews or literature scans and Meta‐analysis of evidence will be excluded on study design criterion.

### 3.9 Settings

The types of setting include: high school, tertiary education institutions, training centres, firms, and any other identified when coding studies.

### 3.10 Search strategy

We propose an elaborate search strategy, to be developed from the entire coding sheet containing filters (i.e., population demographics and socioeconomic characteristics); selected study designs; interventions; and outcomes (Table 5). For instance, impact evaluation‐based study designs will be included in the search terms, since the focus of the map is effectiveness of youth employment interventions.

A standardised search strategy provided in Supporting Information Annex 3 will be used to search over 20 databases, in English (Table 4). While scholarly databases will identify peer reviewed articles, databases for grey literature will mainly provide evaluation reports and working papers. Registers of prospective systematic reviews such as PROSPERO (Table 4) will be searched for on‐going studies. All identified studies will be screened on title and abstract as well as on full text. Completed but unpublished or studies with midterm outcomes will be included in the map.

**Table 4:** Databases

*Scholarly databases*
ERIC: https://eric.ed.gov/
JSTOR: https://www.jstor.org/
ELDIS: https://www.eldis.org/
Wiley Online Library: http://onlinelibrary.wiley.com/
Google Scholar: https://scholar.google.com/
Econlit: https://www.aeaweb.org/econlit/
Web of Science – (Social Science Citation Index and Science Citation Index): https://clarivate.com/webofsciencegroup/solutions/webofscience-ssci/
CAB Global Health (Ovid): https://www.ovid.com/product-details.30.html
CAB Abstracts (Ovid): https://www.wolterskluwer.com/en/solutions/ovid/cab-abstracts-31
REPEC: https://ideas.repec.org/
*University of Chicago Journals*: https://www.journals.uchicago.edu/
*Grey literature sources and registers*
3ie‐ Impact evaluations: https://www.3ieimpact.org/sitewide-search?search_api_fulltext=&sort_by=search_api_relevance
3ie Database of Systematic Reviews: http://www.3ieimpact.org/evidence/systematic-reviews/
3ie Registry for International Development Impact Evaluations: (RIDIE) http://ridie.3ieimpact.org/
USAID ‐ Development Experience Clearinghouse: https://dec.usaid.gov/
SSRN (Social Science Research Network): http://www.ssrn.com/
World Bank Labor Markets: http://www.worldbank.org/labormarkets
World Bank e‐library: https://elibrary.worldbank.org/
Institute for the Study of Labor: http://www.iza.org
Campbell Collaboration: https://campbellcollaboration.org/
IBSS (International Bibliography of the Social Sciences): https://about.proquest.com/en/products-services/ibss-set-c/
Research for Development (DFID's outputs d/base for funded projects) ‐ https://www.gov.uk/dfid-research-outputs
Institute for the Study of Labour (IZA): http://www.iza.org
EPPI CENTRE: https://eppi.ioe.ac.uk/cms/Default.aspx?tabid=185
UNDP International Policy Centre for Inclusive Growth (IPC‐IG): http://www.ipc-undp.org/
International Labour Organization: https://www.ilo.org/Search5/search.do
PROSPERO: https://www.crd.york.ac.uk/prospero/

The rationale for limiting the strategy to English language papers/documents is based on the consideration that the vast majority of studies are potentially written in English and, translation from other languages to English would be compromise by comprehension limitations. The confinement of publication period to two decades (2000–2019) is informed by an observation that development interventions are often best appreciated and understood in a contemporary context (Mawn et al., 2017).

Two information science specialists will conduct study search and internal peer review of their work to avoid errors.

In addition, we will search within eligible systematic reviews and existing maps on youth employment (described earlier under the heading “Existing EGMs on youth employment interventions”). Relevant individuals and organisations will be contacted to access full articles that may not be accessible online. Due to the anticipated big number of studies being included in the map, we will do targeted search of reference lists of included studies through snowballing.

Snowballing and citation tracking will be conducted after studies identified by the standardised search strategy have been coded, meaning that these two activities will be conducted towards the end of the project. While snowballing involves reference tracking the 20 most recent publications, citation tracking will be limited to the 10 most current publications. Snowballing involves searching the reference lists of candidate papers and identifying studies that meet the eligibility criteria of the study. Citation will be conducted in “Google Scholar” by pasting the “reference text” of each study in google scholar search area to show a list of studies which have cited by the candidate study.

### 3.11 Screening of studies

Screening of studies will be carried out in EPPI‐Reviewer 4, which is a web‐based software program for managing and analyzing data in literature reviews. The study references identified from databases searched, will be imported into EPPI‐Reviewer where duplicates will be removed before screening. For studies identified through searching reference lists of systematic reviews and snowballing, bibliographic information will be manually captured in EPPI Reviewer.

Studies will be screened using a five criteria screening tool developed by Dr Howard White, an expert in impact evaluation and evidence synthesis. Included studies shall be written in the English language, should have been published or made available after January 1st, 2000, and by December 31st, 2019, describe an effectiveness study or report quantitative data, describe an intervention for youth between 15 and 35 years and, should be a systematic review or an impact evaluation (Figure 1).

There shall be two levels of screening studies, on the basis of titles and abstracts and on full texts. At first level, titles and abstracts will be screened independently by each of the two reviewers against the inclusion criteria. A reconciliation report comparing the results of the two reviewers will be generated from EPPI Reviewer to identify disagreements which will be resolved through discussion by the reviewers. To add rigour, where the two reviewers do not reach consensus the matter will be forwarded to the third reviewer. At second level, full text papers will again be screened by two reviewers independently and disagreements reconciled through discussion as in the first level.

The screening tool will be piloted through a number of sessions with each of the sessions using about 100 studies. In the first session reviewers will be trained at Africa Centre for Systematic Reviews and Knowledge Translations, College of Health Sciences—Makerere University in Uganda, by Dr. Ashrita Saran, a methods expert.

### 3.12 Data extraction/coding and management

The studies shall be coded on the basis of the information contained in the coding sheet (Table 5). Guidance will be provided for reviewers involved in coding the studies, through piloting coding and checklists. For instance, reviewers will use the most current World Bank classification of countries by income level to code the World Bank Regions. A dictionary defining interventions and outcomes will be provided for reviewers involved in coding studies.

**Table 5:** Coding sheet

**Bibliographic Information**	
	Title
	Author(s)
	Month
	Year
	Publication Type
	Abstract
	Journal title or Report Series
	URL
	Volume
	Publisher
	DOI
	Short title [First author (date)]
	Edition
	ISBN/ISSN
	Issue
	Institution

**Filters**	
Publication status	Complete
	On‐going
Region of the World	Sub‐Saharan Africa
	Latin America and Caribbean
	East Asia and Pacific
	South Asia
	Europe and Central Asia
	North America
World Bank Region	Low‐income countries
	Lower middle income
	Upper middle income
	High income
Population	Youth aged 15–19
	Youth aged 20–24
	Youth aged 25–29
	Youth aged 30–35
	Age not reported
	Male
	Female
	Gender not reported
	Urban
	Rural
	Rural/urban not reported
	Youth with disabilities
	Youth in fragility, conflict, and violence (FCV) contexts
	Youth from disadvantaged background (low‐income families or low education)
	Criminal background
	Ethnic minority
	Humanitarian settings
Sectors	Agriculture
	Services
	Industry: Nonmanufacturing (construction, mining, quarrying, electricity, gas and water supply)
	Industry: Manufacturing
	Sector not reported or not–relevant
Implementer (specify)	NGO, multilateral, government, researcher, private sector (specify in this open field)
Size of the intervention	Number and units in the programme (firm or individual or school in the programme not necessarily only those sampled for the study)
Cost	Cost of intervention
Setting for the intervention	High school
	Tertiary education
	Training centre
	Firm
	Other (specify)
Study design	Experimental designs (i.e., RCTs including cluster randomisation)
	Nonexperimental matching designs (i.e., RDD, PSM, DID/CBA, ITS)
	Regression‐based approaches (i.e., IVs; excludes RDD)

**Interventions**	
Training, up‐skilling and retraining/re‐skilling	Prior Learning Assessment and Recognition (PLAR)
	Education, technical and vocational training (TVET)
	Internship and apprenticeship
	Training centre accreditation and certification
	Training of trainers and teachers
	Business skills training
	Life skills training
Support to employment	Employee Mentoring (Work integrated learning; on job training)
	Career offices/advisory services/career days
	Programme for overseas employment
	Public work programs
	Support to employee mobility and placements
	Wage subsidies
Decent work policies	Labour standards
	Social protection and social security
	Accountability systems
Information	Labour market information
	Digital services and SMS coaching
	Social media campaigns and awareness campaigns
	Value chain development
	Access to services and markets (value chains)
Entrepreneurship promotion and financing	Small and medium sized Enterprise finance (SME)
	Microfinance (to individuals)
	Social impact bonds
	Crowd funding
	Loan guarantees
	Grants
	Self‐financing groups
	Micro‐franchising

**Outcomes**	
Category	Subcategory
Economic	Costs
	Cost Benefit
	Cost effectiveness
	Multiplier, displacement and spillover effects (Effects not directly in the programme, e.g., youth spending earnings to improve local commerce, job displacement)
Education and skills	Education completion and qualifications
	Access to/in education
	Education quality
	Technical skills and vocational training
	Digital skills
	Transferable skills (including life and social skills, e.g., networking, negotiation)
Entrepreneurship	Access to financial services
	Business creation
	Business performance
	Job creation (Jobs for other people, e.g., number of employees)
Employment	Vacancies
	Actively seeking employment
	Employment expectation
	Employment status (including duration)
	Employment consistent with education/training
	Hours worked
	Job quality (include formal vs. informal here)
	Earnings and salary
Welfare	Economic outcomes (except earnings). This also includes income at household level
	Criminal and delinquent behaviour (antisocial behavior)
	Citizenship, values and social behavior [Social behavior is such things as taking part in community activities (clarifying to distinguish from antisocial behavior). Social behavior: alcohol/drugs, hanging out with friends].
	Family health and education
	Inclusion and empowerment (social network). (Engagement in community activities is here [not social behaviour])

Reviewers will pilot the coding sheet before full scale coding of studies. The plan is to have several pilot coding sessions where in each case studies will be independently coded by a pair of reviewers. After each pilot session, the entire EGM team will discuss the results of pilot coding to humanize the application of the coding sheet. Post pre‐test coding of studies will be conducted again by pairs of reviewers who will reconcile disagreements through discussion. Where the two coders do not reach consensus, the mater will be forwarded to the third reviewer/tie breaker.

### 3.13 Quality appraisal

Critical appraisal of each study (impact evaluations and systematic reviews) will be conducted independently by a pair of reviewers who will follow the same procedure used at screening and coding phases, to reconcile disagreements (discussion and help of a tie breaker).

Impact evaluation studies will be assessed using the “Quality assessment of Impact Evaluations” tool developed by Dr. Howard White and Dr. Saran Ashrita. The tool is a checklist of seven items with additional guidance on rating items, expressed as: high confidence, medium confidence or low confidence. However, of the seven items only four (study design (potential confounders taken into account); level of attrition or losses to follow up; definition of outcomes; and baseline balance reports), are the most critical for making decisions.

“A Measurement Tool to Assess systematic Reviews (AMSTAR 2)” will be used to conduct critical appraisal of systematic reviews. AMSTAR has been developed to facilitate the development of high‐quality reviews by guiding conduct and evaluation of reviews. The AMSTAR 2 checklist^3^ contains 16 items, each with concise sentence questions having supplementary guidance on selecting response options (expressed as: “yes”, partial yes and “no”). Overall, the AMSTAR 2 tool rates confidence in components of a systematic review as; High: no or one no‐critical weakness, Moderate: more than one noncritical weakness, Low: one critical flaw with or without noncritical weaknesses and, Critically low: more than one critical flaw with or without noncritical weaknesses (Shea et al., 2017).

## 4 ANALYSIS AND PRESENTATION

### 4.1 Analysis for the narrative report

The unit of analysis will be individual studies where every entry represents a combination of interventions and outcomes. The narrative report of the map findings is a descriptive analysis of the distribution of studies. The report will provide tables representing the number of studies by study design, regions of world, settings, interventions and outcomes. The aggregate map will be presented in a coloured table showing the number of studies by intervention category and outcome domains. A cell will be coloured green for well evidenced if there are over 50 studies, orange for moderately evidenced for 20–49 studies, and red for weakly evidenced if there are fewer than 20 studies. A proposed report outline is provided in Box 1.

Box 1 **Report outline for the youth employment evidence and gap map narrative report**

**Table:** 

Executive summary
Background
Scope of the Youth Employment Evidence and Gap Map Rationale for developing the Youth employment evidence and gap map
Objectives
Methodology
Development of the Framework for the Youth Employment Evidence and Gap Map Search strategy and status of studies Criteria for including and excluding studies Screening and selection of studies Data extraction, coding and management Quality appraisal Analysis
Presentation of findings
Description of the study flow (PRISMA Flow Diagram) Synthesis of included studies Discussion
Strengths and limitation in relation to existing gap maps
Conclusions Cited references
Evidence and gap map authors
Potential conflicts of interest
Annexes

## 5 PRESENTATION OF THE YOUTH EMPLOYMENT EGM

The youth employment EGM will be a matrix, populated with studies that provide evidence for each cell's outcome and intervention combination. Each study will be placed in each cell for which the study provides evidence. That means, majority of studies will appear in the map multiple times because they have multiple outcomes and interventions. Each study will be tied to a weblink which directs the user of the map to an online database where the full text or paper of the study is uploaded. The map will have primary and secondary dimensions which will provide an appealing user‐friendly content visualisation.

The primary dimensions of the map will be interventions (in rows) and outcomes (in columns), presented in a matrix. Interventions will be grouped into categories, subcategories. The outcomes will be arranged in five categories: education and skills, entrepreneurship, employment, welfare and economic. For instance, the “economic” category of outcomes will contain the following outcome subcategories: Cost, cost benefit, cost effectiveness and multiplier effects. In the case of interventions, the “training” category will comprise; TVET; internship and apprenticeship; Training centre accreditation and certification; training of trainers and teachers; business skills training; and life skills training.

The map will be interactive, through inspection of bubbles and cells of the matrix. By pointing the cursor at the bubble, the user can see the number of studies in that cell. Clicking on a cell displays a list of studies in that cell. Clicking on a row or column heading displays the list of studies in that row or column. Single studies and systematic reviews will be differentiated by colours of the bubbles.

At the bottom of the map, the colour‐coding of bubbles will represent study quality based on critical appraisal of included studies. Study quality ratings include: low quality impact evaluation; medium and high‐quality impact evaluation; low quality systematic review; or medium and high‐quality systematic review. Pointing the cursor at the cell will identify single studies from systematic reviews, according to study quality rating.

The secondary dimension of the map will be the taskbar menu (Filters, About and View records), which help a user to navigate the map. Filters are search aids which will help the user of the map to quickly find records matching criteria of interest. The filters include study design, regions of the world, country and population subgroups. The population subgroups will include youth with disabilities, youth in FCV contexts and, youth from disadvantaged background (low‐income families or low education); criminal background; ethnic minorities and; humanitarian settings. Clicking on the “About” tab in the taskbar menu will display the “about” text which describes the map. Clicking on the “View Records” tab will display a record of all studies in the map, offering the user options to directly access the full texts (pdfs.) of the displayed records as well as exporting the reference list which is compatible with other reference management software such as Endnote.

## ROLES AND RESPONSIBILITIES

***Project Director:*** Ekwaro A. Obuku has an extensive experience in research, evidence synthesis, policy advocacy, training and teaching. As the Director of Africa Centre for Systematic Reviews and Knowledge Translation, Ekwaro has been key in building capacity for evidence synthesis and translation of research to policy through engagements with scientists, policy makers and decision takers across sub‐Saharan Africa. As President of the Uganda Medical Association, Ekwaro is at the forefront of policy engagement operating at all levels including Office of the Prime Minister and the Presidency. He is a coauthor of several systematic reviews and tutors Systematic Reviews at Makerere University. He recently started a course in Evidence Synthesis for Masters Students in Clinical Epidemiology and Biostatistics at Makerere University, Uganda.

***Methods expertise:*** Ashrita Saran and Howard White have previous experience in systematic review methodology, including searching, data collection, and theory‐based synthesis, which means they are proficient in carrying out the various processes in an EGM, such as search, eligibility screening, quality assessment and coding. They have undertaken an overview of approaches to mapping in a range of organization.

***Content**:* Howard White is a development economist who has previously studied labour markets in Africa. As described below, the International Labour Organization (ILO) coauthors will act as advisors.

***Coders:*** Robert Apunyo is an experienced screener and coder who has previously worked on three EGMs supported by the Campbell Collaboration. Robert has attended training workshops on evidence synthesis provided by Cochrane South Africa, Campbell Collaboration and, Africa Centre for Systemic Reviews and Knowledge Translation. Caroline Otike is an experienced screener and coder who has previously worked on Campbell Collaboration supported EGMs. Thomas Katairo has attended trainings in systematic reviews provided by the Africa Centre.

***Information retrieval expertise:*** Alison Annet Kinengyere and John Eyres will conduct information retrieval. Alison is an information science pecialist with vast skills in designing and implementing search strategies. She has supported production of many systematic reviews.

***Advisors:*** Sussana Puerto and Drew Gardiner will be Senior Advisors, content and policy on youth employment.

## TIMEFRAME

1. Publication of the protocol: 2020.
2. Search and screening: 2020.
3. Draft EGM: 2020.
4. Submission of the youth evidence and gap map and a narrative report to Mastercard Foundation will be in December 2020.

## FUNDING

The funding for the map is from by Mastercard Foundation.

## POTENTIAL CONFLICTS OF INTEREST

No conflict of interest.

## Supporting information

Supporting information.

## Footnotes

^1^
“Youth employment EGM” is the short title adopted from the main title, “interventions to increase Youth employment: An evidence and gap map”.

^2^

https://stats.oecd.org/glossary/detail.asp?ID=28ALMPs include all public expenditure (other than education) whose objective is to improve prospects of beneficiaries in finding employment or increase their earnings capacity. In other words, ALMPs are public programmes that intervene in the labour market to help the unemployed find work.

^3^

http://www.amstar.ca/Amstar_Checklist.php.
