# Supplementary material for: PROTOCOL: Interventions to increase youth employment: An evidence and gap map
Source: Campbell Syst Rev. 2021 Oct 18;17(4):e1196. doi: 10.1002/cl2.1196 (PMC8988741; doi:10.1002/cl2.1196)
Supplement: Supplementary file 1 — Supporting information. [file CL2-17-e1196-s001.docx]

**Annex: 1: Definitions of inerventions**

| **Intervention category** | **Intervention sub-category** |
| --- | --- |
| **Training, up-skilling and retraining / re-skilling:** Training is teaching, or developing in oneself or others, any skills and knowledge or fitness that relate to specific useful competencies. Up-skilling-the process of learning new skills or of teaching workers new skills (Cambridge) Retraining is a practice employer may require for their workers to make them learn new skills especially to avoid stagnant workforce. | **Prior Learning Assessment and Recognition (PLAR):** Prior learning assessment and recognition defines processes that allow individuals to identify, document, have assessed and gain recognition for their prior learning. The learning may be formal, informal, non-formal, or experiential. The context of the learning is not key to the process as the focus is on the learning. PLAR processes can be undertaken for several purposes, including self-knowledge, credit or advanced standing at an academic institution, for employment, licensure, career planning or recruitment. Tools such as challenge exams, demonstrations, structured interviews, simulations and portfolios can be used alone or in combination, for experiential learning and competency assessment in such instances (CAPLA,^[[1]](#footnote-1)^ 2019). |
|  | **Technical and vocational training (TVET):** Technical and vocational education and training’ (TVET) is understood as comprising education, training and skills development relating to a wide range of occupational fields, production, services and livelihoods. TVET, as part of lifelong learning, can take place at secondary, post-secondary and tertiary levels and includes work-based learning and continuing training and professional development, which may lead to qualifications. TVET also includes a wide range of skills development opportunities attuned to national and local contexts. Learning to learn, the development of literacy and numeracy skills, transversal skills and citizenship skills are integral components of TVET (UNESCO, 2019a). Stipends during training should be coded here. |
|  | **Internship:**  These are short-term periods of temporary work experience, typically lasting for a few weeks or months mainly to gain on-the-job experience of working in a particular role, organization or industry sector *(*Finch, (2018) & International Labour Organization, (2012*).*  **Apprenticeship**: An apprenticeship is a formal employment program that provides training to do a specific job. Unlike internships, apprenticeships employ people who already know which career path they wish to follow. If they join a program, they will sign a contract with the employer and learn specific skills during their apprenticeship. Training last longer – a few years (Finch, 2018). |
|  | **Training centre accreditation and certification:**  **Accreditation** is the process by which a (non) governmental or private body evaluates the quality of a higher education institution as a whole or of a specific educational programme in order to formally recognize it as having met certain predetermined minimal criteria or standards. Types of accreditation include Institutional, Regional and Specialized accreditation (Vlăsceanu *et al.,* 2004).  **Certification** is the process by which an agency or an association acknowledges the achievement of established quality standards and usually grants certain privileges or written assurance to the target individual – student (Vlăsceanu *et al.,* 2004). |
|  | **Training of trainers and teachers:** Training of Trainers (TOT) is a high-level professional learning process for qualified trainers who provide training and capacity-building assistance (Program Success Centre for Sexual & Reproductive Health, 2019) e.g., on youth employment skills. An example is the TOTs on youth empowerment for advocacy and campaigning on youth unemployment. |
|  | **Business skills training:** This involves training in Financial management, Marketing, sales and customer service, Leadership, Project management and planning, Delegation and time management, Problem solving and, Networking (Small Business Development Corporation, 2019). |
|  | **Life skills training:** Life skills training is intended to strengthen trainees’ self-esteem and work habits by equipping them with a wide set of behaviours, attitudes, and personal qualities so that they develop the ability to navigate their environment, work well with others, perform well, and achieve set goals. Examples life skills or soft skills include social skills, communication skills, problem solving skills, decision making skills and elf-control (Lippman et al., 2015). Life skills training is deferent from vocational training in that the former is meant to address the technical training needs of local employers (Ibarraran, et al., 2012). |
| Support to employment | **Employee Mentoring (Work integrated learning, On-job-training (OJT):** The relevant/applicable meaning of OJT is: a Work Based Training or In-service Training or On-Site Training (UNESCO, 2019b) for employees. It excludes apprentices and interns. |
|  | **Career offices/advisory services/career days:** Career centres/offices provide information or assist individuals in making and implementing informed educational and occupational choices. A career guidance and counselling program develops an individual's competencies in self-knowledge, educational and occupational exploration and career planning (U.S.A. Department of Education, 2019). |
|  | **Programme for overseas employment:** Overseas employment refers to employment of a migrant worker in a foreign country, where workers often sign agreements with their employers for a specified period of time. Overseas employment programmes are mainly set up and run government agencies of developing countries in attempt to address high rates of unemployment at home buy exporting labour to foreign countries. Philippines and Jamaica provide some of the most elaborate examples of overseas employment programmes. |
|  | **Public work programs:** Public works in general means construction, alteration, demolition, installation, or repair work done under contract and paid in whole or in part out of public funds (USA. State of California Department of Industrial Relations 2020). Primary beneficiaries of public works programs are the hard-to-employ and socially vulnerable groups who, by being engaged in public works, are offered a chance to earn regular income, leastwise in a short run, and maintain and improve their skills (EU, 2017). |
|  | **Support to employee mobility and placements:** Including financial assistance for job search, job search assistance.  Geographical mobility (employee relocation); Occupational mobility (employee movement into and out of different job species), Organizational mobility (movement of individuals up and down or laterally amongst organization) (Scism, 1974).  A job placement is when an [unemployed](https://www.ldoceonline.com/dictionary/unemployed) person, is put into a job that is [suitable](https://www.ldoceonline.com/dictionary/suitable) to their [skills](https://www.ldoceonline.com/dictionary/skill) and [interests](https://www.ldoceonline.com/dictionary/interest), usually for a [temporary](https://www.ldoceonline.com/dictionary/temporary) [period](https://www.ldoceonline.com/dictionary/period) (Longman, 2019)**.** |
|  | **Wage subsidies:** These are transfers on non-wage employment costs. Their main goal is to provide incentives for employers to hire members of the target group such as unemployed youth (Bordos *et al.,* 2015). Governments are the major providers of wage subsidies. These could be direct money transfers to firms or tax deductions for firms employing youth. |
| Decent work Policies (Policy reforms Influencing professional practices & Shaping policy for data sharing).  Decent work sums up the aspirations of people in their working lives. It involves opportunities for work that is productive and delivers fair income, security in the workplace and social protection for families, better prospects for personal development and social integration (International Labour Organization, 2019). | **Labour standards:** These are legal instruments drawn up by the ILO's constituents (governments, employers and workers) and setting out basic principles and rights at work. They are either Conventions (or Protocols), which are legally binding international treaties that may be ratified by member states, or Recommendations, which serve as non-binding guidelines. In many cases, a Convention lays down the basic principles to be implemented by ratifying countries, while a related Recommendation supplements the Convention by providing more detailed guidelines on how it could be applied. Recommendations can also be autonomous, i.e., not linked to a Convention (International Labour Organization, 2019). |
|  | **Social protection and social security:** Social protection includes benefits for unemployment, employment injury etc. Social protection systems address all policy areas by a mix of contributory schemes (***social insurance***) and non-contributory tax-financed benefits, including ***social assistance*** (International Labour Organization, 2017). **Social security** involves access to health care and income security, particularly in cases of unemployment, work injury, maternity or loss of a main income earner (International Labour Organization, 2020). |
|  | **Accountability systems:** Accountability is the obligation to demonstrate that policy has been designed and implemented effectively, and to report on results in a timely and accurate manner. The employment policy accountability system generally comprises seven elements: (a) national level priorities with goals and targets, (b) work plans with specific targets and measures, (c) a budget allocation, (d) a performance framework with delivery plan and indicators of outcome, (e) statistics and reporting, (f) monitoring and evaluation, and (g) adjustment and improvement of employment policy (International Labour Organization, n.d.). |
|  |  |
| Information | **Labour market information:** Labour market information covers the principal elements of the labour market and its operations. For instance, labour market information on current demand, broken down by occupation and skills level, including early identification of sectoral trends and of changes in technology and occupations leading to changing skills composition (International Labour Organization, 2011) |
|  | **Digital services and SMS coaching:** Digital government services (also called e-government) are defined as service delivery within government — as well as between government and the public — using information and communication technologies. Common digital services range from filling tax returns to renewing a driver’s license to applying for a pet license. Nearly any government form or service can be offered digitally (Granicus, 2019). |
|  | **Social media campaigns and awareness campaigns:** Social media campaigns are marketing campaigns on platforms like Facebook, Twitter, Linkedin or Instagram. They have the potential to engage followers, boost brand awareness e.g., on matters of youth employment ([Heavey](https://thrivehive.com/author/dheavey/), 2017). |
|  | **Access to services and markets (value chains):** Market access in services is more complex than market access for trade in goods. For trade in goods, market access is about reducing mainly border measures such as tariffs that are imposed on goods as they enter a market. For trade in services, market access is about reducing government policy interventions, which are less visible and may be applied after a service supplier has entered the market. These measures take the form of government regulations that are usually aimed at domestic policy objectives rather than trade policy objectives (McGuire, 2002). |
|  | **Value chain development:** The ILO Value Chain Development approach looks at market dynamics and relationships between the different actors in the chain with the objective of strengthening the whole market system - enterprises, business relationships, financial networks, supporting functions, rules and norms, and the business environment – in a way that ensures greater benefits for the poor from economic growth and development (Nutz & Sievers, 2015). |
| Entrepreneurship promotion and Financing | Small and medium sized Enterprise finance (SME): Improving SMEs’ access to finance and finding innovative solutions to unlock sources of capital (World Bank 2020). This may include lending operations or organizations/agencies and countries setting a side specific funding for SMEs. |
|  |  |
|  |  |
|  | **Microfinance (to individuals):** Microfinance, also called [microcredit](https://www.investopedia.com/terms/m/microcredit.asp)​, is a type of banking service provided to unemployed or low-income individuals or groups who otherwise would have no other access to financial services (Investopedia, 2019b). |
|  | **Social impact bonds:** A social impact bond (SIB) is a contract with the public sector or governing authority, whereby it pays for better social outcomes in certain areas, such as youth employment, and passes on the part of the savings achieved to investors (Investopedia, 2019). Implementers are paid for results (youth employed) not activities. |
|  | **Crowd funding**: Crowdfunding is the use of small amounts of capital from a large number of individuals to finance a new business venture. Crowdfunding makes use of the easy accessibility of vast networks of people through [social media](https://www.investopedia.com/terms/s/social-media.asp) and crowdfunding websites to bring investors and entrepreneurs together, with the potential to increase entrepreneurship by expanding the pool of investors beyond the traditional circle of owners, relatives and [venture capitalists](https://www.investopedia.com/terms/v/venturecapitalist.asp) (Investopedia, 2019a). |
|  | **Loan guarantees:** A guaranteed loan is a loan that a third party (e.g., government agency) guarantees – or assumes the debt obligation for – in the event that the borrower [defaults](https://www.investopedia.com/terms/d/default2.asp). Guaranteed loan agreements may be made when borrowers such as unemployed youth are considered unattractive candidates for regular bank loans. It is a way for people (unemployed youth) who need financial assistance to secure funds when they otherwise may not qualify to acquire them (Kagan, 2019). |
|  | **Grants:** These are funds that do not have to be paid back by the recipients, under most conditions. Grants meant for youth employment can include; entrepreneurship grants, education grants and research money. Some **grants** have waiting periods, called lock-up or vesting periods, before the grantee can take full ownership of the financial reward (Chen, 2018). |
|  | **Self-financing groups:** Self-Help or financing groups are small groups, often mostly or only women, who save a certain amount of money each week or each month and have group meetings weekly or monthly. Out of these collective savings they issue each other loans. Groups decide democratically how much interest they will charge, what the penalties will be for not coming to the meetings or showing up late to the meetings, and what amount they will save each week or month. They decide what the requirements are for dropping out of the group and the requirements for new members joining (Flynn, 2013). |
|  | **Micro franchising**: Micro-franchising is a business model that applies traditional franchising to very small businesses. It is a systemized approach to replicating micro-enterprises like drive-in coffee kiosks, mall products and services, food stands, and just about any other type of business that sells low-cost products or services, primarily in high traffic areas (Forbes, 2019). |

**Annex 2: Definitions of Outcomes**

| **Economic outcomes:**  These include cost, cost benefits, cost effectiveness and, multiplier and spillover effects. |
| --- |
| **Cost:** Costs are the necessary expenditures that have to be paid or given up in order to get something (Business Dictionary 2020) or for implementation of a youth employment intervention. For example, cost/monetary value of wage subsidy (Betcherman, et al 2010). |
| **Cost Benefits:** Cost Benefit Analysis is an economic evaluation technique that compares the cost of the intervention with the benefit incurred, where the benefit is measure by monetary unit. Here, both costs and consequences are measured in monetary unit. Net Benefit= Benefits – Costs |
| **Cost effectiveness:** Cost Effectiveness Analysis (CEA) is an economic evaluation technique that compares ‘cost per consequence’ of two or more interventions, where the consequences are measured by ‘natural’ units (life years gained, saved years of life). CEA focuses on non-monetary outcomes. Cost effectiveness ratio (CER) = Cost of Intervention/Effect of Intervention (Cellini& Kee, 2015) |
| **Multiplier and spillover effects:** Effects not directly in the programme e.g., youth spending earnings to improve local commerce and displacement of employment. |
| **Education and skills outcomes:**  Educational outcomes broadly refer to notable achievements in education, satisfaction with quality of education programs; transformative experiences and, career readiness and performance (Fleming, 2015). Specific outcomes defined below include (education completion, education quality and access to education).  The ‘Skills set’ of outcomes in this context include attained technical and transferable skills. Technical skills refer to those got after undergoing a training and such skills are meant to address the technical training needs of local employers (Ibarraran, et al., 2012). However, transferable skills include life and social skills such as networking skills, are meant help the individual navigate their environment. |
| **Education completion:** Percentage of a cohort of children or young people aged 3-5 years above the intended age for the last grade of each level of education who have completed that grade. The intended age for the last grade of each level of education is the age at which pupils would enter the grade if they had started school at the official primary entrance age, had studied full-time and had progressed without repeating or skipping a grade. For example, if the official age of entry into primary education is 6 years, and if primary education has 6 grades, the intended age for the last grade of primary education is 11 years. In this case, 14-16 years (11 + 3 = 14 and 11 + 5 = 16) would be the reference age group for calculation of the primary completion rate (UNESCO, 2019c). |
| **Access to education:** Access to education includes: on-schedule enrolment and progression at an appropriate age, regular attendance, learning consistent with national achievement norms, a learning environment that is safe enough to allow learning to take place, and opportunities to learn that are equitably distributed (Lewin, 2015). |
| **Education quality:** A good quality education is one that provides all learners with capabilities they require to become economically productive, develop sustainable livelihoods, contribute to peaceful and democratic societies and enhance individual well-being. The learning outcomes that are required vary according to context but at the end of the basic education cycle must include threshold levels of literacy and numeracy, basic scientific knowledge and life skills including awareness and prevention of disease. Capacity development to improve the quality of teachers and other education stakeholders is crucial throughout this process (vvob, 2019). |
| **Technical skills and vocational training (TVET)**: Technical and vocational education and training’ (TVET) is understood as comprising education, training and skills development relating to a wide range of occupational fields, production, services and livelihoods. TVET, as part of lifelong learning, can take place at secondary, post-secondary and tertiary levels and includes work-based learning and continuing training and professional development, which may lead to qualifications. TVET also includes a wide range of skills development opportunities attuned to national and local contexts. Learning to learn, the development of literacy and numeracy skills, transversal skills and citizenship skills are integral components of TVET (UNESCO, 2019d). |
| **Digital skills:** Digital skills are defined as a range of abilities to use digital devices, communication applications, and networks to access and manage information. They enable people to create and share digital content, communicate and collaborate, and solve problems for effective and creative self-fulfillment in life, learning, work, and social activities at large (UNESCO 2019e). |
| **Transferable skills (including life skills e.g., networking, negotiation):** A transferable skill is a skill learned one another context (in the case of research) and used in another (for example future employment whether in research, business etc.). They can serve as a bridge from study to or from one career to another as they enable the subject and related skills to be applied and developed effectively in different work environments. They include skills such as communication skills and organizational skills (Organisation for Economic Co-operation and Development (OECD), 2012). |
| **Entrepreneurship outcomes:**  These broadly refer to business creation, business performance, job creation as well as access to financial services. |
| **Access to financial services:** The availability to a given person of affordable and appropriate financial services e.g., to youth investors. Access is often seen as the goal of financial inclusion (Centre for Financial Inclusion, 2019). |
| **Business creation:** Creating a new business is a process which starts with an idea and involves many stages before launching a new product or process on the market (Innovaccess - Intellectual Property Portal, 2019) |
| **Business performance:** Including profits, sales, number of employees and jobs created, capital and investment, business creation and business survival ([Kluve](https://onlinelibrary.wiley.com/action/doSearch?ContribAuthorStored=Kluve%2C+Jochen), *et al* 2014). |
| **Job creation:** The process by which the number of jobs in an economy increases. Job creation often refers to government policies intended to reduce unemployment. Job creation programs may take a variety of forms. For example, a government may lower taxes and reduce regulation to make hiring less expensive. On the other hand, a government may hire workers itself, for example, to build a road (Farlex Financial Dictionary, 2012) |
| **Employment outcomes:**  These refer to the following: Employment status, seeking employment, vacancies, employment expectation, employment consistent with education/training, Hours worked, job quality and earnings. |
| **Vacancies:** Number of unoccupied positions for a job. |
| **Actively seeking employment:** This includes actions undertaken by a youth during participation in a youth employment programme, in attempt to find employment: job applications submitted; attendance of job fairs, reviewing job advertisements and, attending job interviews etc. (Azevedo, et al., 2013). |
| **Employment expectation:** This may entail expectations of improved future employment conditions (Acevedo, 2017), likelihood of being offered job and, likelihood of being deployed if offered a job (Beam, 2016). |
| **Employment status (including duration):** **Employment status** is the status of a worker in a company on the basis of the contract of work or duration of work done. A worker may be a full-time employee, part-time employee, or an employee on a casual basis or unemployed. S/he could be employed temporarily for a specific project only, or on a permanent basis. Part-time wage labor could combine with part-time self-employment. The worker could be employed also as an apprentice (US Legal, 2019).  **Employment duration:** Longevity; duration of service or employment. Often used to indicate how long an employee has worked at a company or an individual has belonged to an organization (Business Directory, 2019). |
| **Employment consistent with education/training:** This refers to employment which is related to the skill learned or owned by an employee (Ahmed et al., 2014). Skills match with tasks or worker is not under/over qualified for the job (Abebe et al., 2017). |
| **Hours worked:** In general, ‘hours worked’ includes all time an employee must be on duty, or on the employer's premises or at any other prescribed place of work. Also included is any additional time the employee is allowed to work, (i.e., overtime, hourly wage, double time) (USA. Department of Labour, 2019). |
| **Job quality:** Job quality complements measures of job quantity to provide an assessment of employment strategy. Job quality is a multidimensional concept where different policy agenda and disciplines emphasize different dimensions. Job quality is measured at the level of the job. It includes job features captured from an objective perspective, which can be observed and are related to meeting people’s needs from work. It is made up of all the characteristics of work and employment that have been proven to have a causal relationship with health and well-being. Positive and negative features of the jobs are included. These indicators reflect the job resources (physical, psychological, social or organizational aspects) and job demands, or the processes that influence them (Eurofund, 2019). Job quality includes informal vs informal jobs. |
| **Earnings:** money obtained in return for labour or services. Including reported earnings and income, household income, consumption and salary and/or wage ([Kluve](https://onlinelibrary.wiley.com/action/doSearch?ContribAuthorStored=Kluve%2C+Jochen) *et al.,* 2014). |
| **Welfare outcomes:**  These include the following: Criminal and delinquent behavior; citizenship; values and social behavior, family health and education and; Inclusion and empowerment (social network. |
| **Criminal and delinquent behavior (anti-social behavior):** Crime can involve violence, sex or drugs but also discrimination, road rage and burglary. Crime is any behaviour and any act, activity or event that is punishable by law (Government of Netherlands, 2019). |
| **Citizenship, values and social behavior:** Personal values are reliable cross-situational predictors of attitudes and behaviour. Personal values are individual conceptions of the desirable that guide behavior in little things like donating to charity or spending time with the family and in life-defining decisions ([Ponizovskiy](http://www.frontiersin.org/people/u/441564) *et al.,* 2019). Social behaviour is such things as taking part in community activities. |
| **Economic outcomes (except earnings):** These include assets, savings and business profits of firms etc. (Blattman, 2014, Fiala, 2014 & Hirshleifer et al., 2014). It also includes income at household level. |
| **Family health and education:** This includes reproductive health, medical expenses, household nutrition, hygiene, education expenditure etc. (Blattman at al., 2014). |
| **Inclusion and empowerment (social network):** Personal empowerment is about taking control of your own life, and making positive decisions based on what you want (Mind Tools, 2019). Outcomes include but not limited to quality of family relationships, partner relations and autonomy e.g.,' to spend earnings buy without permission from partner (Blattman at al. ,2014). Engagement in community activities should be coded here. |

**Annex 3: Search strategy for the youth employment evidence and gap map**

1. **ERIC DATABASE:** [https://eric.ed.gov/](https://eric.ed.gov/%20)

**With filters: Publication date: Jan 2000-Dec2019; Boolean/phrase/English**

**(Selected Boolean phrase)**

| **Results** | **Type** |
| --- | --- |
| S1 | Train* or retrain* or re-train* or retool* or re-tool* or skill* or educat* or formal or on-the-job or vocational or TVET or OTJ or apprenticeship* or mentor* or internship or upskilling or career or financ* or microfinance or guarantees or policies or policy or micro-franchising or “international labour standard*” or “international labor standard*” or employ*” or ALMPs or “active labour market program*” or “active labor market program*” or “value chain “ or entrepreneur* or cost-effectiveness or "cost per job" or “social protection” or “social security” or “accountability systems” or scholarship* or earn* |
| S2 | Youth* or Teen* or “young people” or “young adult*” or “young person*” or adolescen* or “early adult*” or 'young women' or 'young men' or “aged from 15” or 'over 15 years' or 'under 35 years' |
| 3 | 1 AND 2 |
| 4 | eval* or assess* or analy* or estimate or effect or intervention* or measure* |
| 5 | “random* controlled trial” or “controlled clinical trial” or RCT or “control* trial” or “random allocat*” or “difference in difference*” or “difference-in-difference*” or “double difference*” or “regression discontinuity” or “instrumental variable*” or “propensity score” or quasi-experiment* or “quasi experiment*” or QED or QES or matching or “IV estimation” or “instrumental variable” |
| 6 | “systematic review*” or meta-analy* or “meta analy*” |
| 7 | 4 OR 5 OR 6 |
| 8 | 3 AND 7 |
| 9 | Limiters - Date Published: 20000101-20191231 |
| 10 | Limiters – English language |

1. **JSTOR:** <https://www.jstor.org/>

| **Results** | **Type** |
| --- | --- |
| 1 | ((ti:(train* OR educat* OR mentor* OR job* OR skill* OR lab*r market OR employ* OR job OR empower*) AND ti:(youth* or teen* or "young people" or adolesc*)) AND ti:(eval* or assess* or analys* or "systematic review*" or RCT)) AND la:(eng OR en) |
| 2 | Limiters – Date published: 20000101-20191231 |

1. **3ie- Impact evaluations:** <https://www.3ieimpact.org/sitewide-search?search_api_fulltext=&sort_by=search_api_relevance>

| **Results** | **Type** |
| --- | --- |
| 1 | (youth OR young people) AND (educat* OR train* OR technical or vocational) AND (employment OR “labor market” OR “labour market”) |

1. **3ie Database of Systematic Reviews:** <http://www.3ieimpact.org/evidence/systematic-reviews/>

| **Results** | **Type** |
| --- | --- |
| 1 | (youth OR "young people") AND (educat* OR train* OR technical OR vocational) AND (employ* OR "labor market" OR “labour market ) |

1. **3ie Registry for International Development Impact Evaluations - (RIDIE):** <http://ridie.3ieimpact.org/>

| **Results** | **Type** |
| --- | --- |
| 1 | (youth or "Young people") + (educat* or train* or "technical and vocational education" or entrepreneur*) + (employ* OR "labor market" OR “labour market”) |

1. **USAID - Development Experience Clearinghouse:** <https://dec.usaid.gov/>

| **Results** | **Type** |
| --- | --- |
| 1 | Youth employment |

1. **Google Scholar:** <https://scholar.google.com/>

| **Results** | **Type** |
| --- | --- |
|  | (train* OR educat* OR skill* OR re-tool* OR technical OR vocational OR TVET OR scholarship* OR apprentice*) AND youth* AND (employ* OR empower* OR ALMP OR AND (labour OR labor) OR entrepreneur*) AND (eval* OR “systematic review") |

1. SSRN (Social Science Research Network): <http://www.ssrn.com/>

| **Results** | **Type** |
| --- | --- |
| 1 | youth employment AND training AND evaluation |

1. Wiley Online: <https://onlinelibrary.wiley.com/>

| **Results** | **Type** |
| --- | --- |
| 1 | (train* OR skill* OR educat* OR apprenticeship*) in Title AND (youth* OR "young adult*" OR teen* OR "young people") in Title AND (employment OR "labour market" OR “labor market” OR enterpreneur*) anywhere AND (eval* OR “systematic review” OR RCT) anywhere |
|  | Limiters: 2000-2019 |

1. **University of Chicago Journals:** <https://www.journals.uchicago.edu/>

| **Results** | **Type** |
| --- | --- |
| 1 | (youth* OR "young adult*" OR "young people") AND (employment OR "labour market" OR "labor market") AND (educat* OR train*) in abstract |

1. **World Bank Labor Markets**: <http://www.worldbank.org/labormarkets>

| **Results** | **Type** |
| --- | --- |
| 1 | In keywords: (youth* OR "young adult*" OR "young people" OR teen*) AND In keyword: (employment OR "labour market" OR "labor market") AND In Keyword: (educat* OR train* OR skill*) AND In keyword: (evaluation OR “systematic review”) |

1. IBSS (International Bibliography of the Social Sciences): <https://about.proquest.com/en/products-services/ibss-set-c/>
2. **REPEC & World Bank e-library (through EBSCO Discovery):** <https://econpapers.repec.org/>

| **Results** | **Type** |
| --- | --- |
| S1 | Train* or retrain* or re-train* or retool* or re-tool* or skill* or educat* or formal or on-the-job or vocational or TVET or OTJ or apprenticeship* or mentor* or internship or upskilling or career or financ* or microfinance or guarantees or policies or policy or micro-franchising or “international labour standard*” or “international labor standard*” or employ*” or ALMPs or “active labour market program*” or “active labor market program*” or “value chain “ or entrepreneur* or cost-effectiveness or "cost per job" or “social protection” or “social security” or “accountability systems” or scholarship* or earn* |
| S2 | TI ( (Youth* or Teen* or "young people" or "young adult*" or "young person*" or adolescen* or "early adult*" or "aged from 15" or "aged under 35") ) OR SU ( (Youth* or Teen* or "young people" or "young adult*" or "young person*" or adolescen* or "early adult*" or “young women” or “young men” or "aged from 15" or “over 15 years” or "aged under 35" or ) ) Limiters - Date of Publication: 20000101-20191231  Database - Discovery Service for 3ie, Inc. |
| S3 | TI ( (eval* or assess* or analy* or estimat* or effect or intervention* or measure*) ) OR AB ( (eval* or assess* or analy* or estimat* or effect or intervention* or measure*) ) OR SU ( (eval* or assess* or analy* or estimat* or effect or intervention* or measure*) ) Limiters - Date of Publication: 20000101-20191231  Database - Discovery Service for 3ie, Inc. |
| S4 | TI ( ("random* controlled trial" or "controlled clinical trial" or RCT or "control* trial" or "random* allocat*" or "difference in difference*" or difference-in-difference* or "double difference*" or "regression discontinuity" or "instrumental variable*" or "propensity score" or quasi-experiment* or "quasi experiment*" or QED or QES or matching or "IV estimation") ) OR AB ( ("random* controlled trial" or "controlled clinical trial" or RCT or "control* trial" or "random* allocat*" or "difference in difference*" or difference-in-difference* or "double difference*" or "regression discontinuity" or "instrumental variable*" or "propensity score" or quasi-experiment* or "quasi experiment*" or QED or QES or matching or "IV estimation") ) OR SU ( ("random* controlled trial" or "controlled clinical trial" or RCT or "control* trial" or "random* allocat*" or "difference in difference*" or difference-in-difference* or "double difference*" or "regression discontinuity" or "instrumental variable*" or "propensity score" or quasi-experiment* or "quasi experiment*" or QED or QES or matching or "IV estimation") ) Limiters - Date of Publication: 20000101-20191231  Database - Discovery Service for 3ie, Inc. |
| S5 | TI ( ("systematic review*" or (systematic* N2 review*) or meta-analy* or "meta analy*") ) OR AB ( ("systematic review*" or (systematic* N2 review*) or meta-analy* or "meta analy*") ) OR SU ( ("systematic review*" or (systematic* N2 review*) or meta-analy* or "meta analy*") ) Limiters - Date of Publication: 20000101-20191231  Database - Discovery Service for 3ie, Inc. |
| S6 | S3 OR S4 OR S5  Database - Discovery Service for 3ie, Inc. |
| S7 | S1 AND S2 AND S6  Database - Discovery Service for 3ie, Inc. |
|  | Final Result limited to Repec |
|  | Final Result limited to World Bank e-library |

1. **Institute for the Study of Labour (IZA):** <http://www.iza.org>

| **Results** | **Type** |
| --- | --- |
| 1 | youth employment AND (evaluation OR systematic review or meta analysis) |

1. **Campbell Collaboration:** <https://www.campbellcollaboration.org/>

| **Results** | **Type** |
| --- | --- |
| 1 | youth AND employment |

1. **EPPI CENTRE:** <https://eppi.ioe.ac.uk/cms/Default.aspx?tabid=185>

| **Results** | **Type** |
| --- | --- |
| 1 | Youth |
| 2 | Teenagers |
| 3 | Young adults |
| 4 | Young people |
| 5 | Young women |
| 6 | Young men |
| 7 | Aged from 15 |
| 8 | Over 15 years |
| 9 | Under 35 years |
| 10 | 1-9/or |
| 11 | Education |
| 12 | skill* |
| 13 | Internship |
| 14 | Upskilling |
| 15 | Career |
| 16 | empower* |
| 17 | Micro-franchising |
| 18 | Cost-effectiveness |
| 19 | Cost per job |
| 20 | train* |
| 21 | 11-20/or |
| 22 | 10 AND 21 |
| 23 | Evaluation |
| 24 | “Systematic review” |
| 25 | 12 OR 13 |
| 20 | 11 AND 14 |

1. **ELDIS:** <https://www.eldis.org/>

| **Results** | **Type** |
| --- | --- |
|  | (Training OR skill* OR educat*) AND Youth AND (employ* OR income) AND evaluation |

19 **Research for Development (DfID’s outputs d/base for funded projects)**: <https://www.gov.uk/dfid-research-outputs>

| **Results** | **Type** |
| --- | --- |
|  | (educat* OR train* OR skill*) AND youth* AND (employment OR "labour market" OR "labor market") AND ("impact evaluation” OR “systematic review”) |

20. UNDP International Policy Centre for Inclusive Growth (IPC-IG): <http://www.ipc-undp.org/>

| **Results** | **Type** |
| --- | --- |
|  | Youth employment |

21. International Labour Organization: <https://www.ilo.org/Search5/search.do>

| **Results** | **Type** |
| --- | --- |
|  | ("youth OR "young people") AND employment AND training AND ("labour market" OR "labor market") AND evaluation |

**22. EconLit:** <https://www.aeaweb.org/econlit/>

| **Results** | **Type** |
| --- | --- |
| 1 | (Train* or retrain* or re-train* or retool* or re-tool* or skill* or educat* or internship or upskilling or career or formal or on-the-job or vocational or TVET or OTJ or apprenticeship* or mentor* or financ* or microfinance or guarantees or policies or policy or "international labour standard*" or "international labor standard*" or employ* or ALMPs or "active labour market*" or "active labor market*" or "value chain" or entrepreneur* or "social protection" or "social security" or (accountab* adj3 system*) or scholarship* or earn*).ti. |
| 2 | I21 or I22 or I26 or J21 or L26 or M53).cc. |
| 3 | or/1-2 |
| 4 | (Youth* or Teen* or "young people" or "young adult*" or "young person*" or adolescen* or "early adult*" or "aged from 15" or "aged under 35").ti,kw. |
| 5 | J13.cc. |
| 6 | or/4-5 |
| 7 | eval* or assess* or analy* or estimat* or effect or intervention* or measure*).ti,ab. |
| 8 | ("random* controlled trial" or "controlled clinical trial" or RCT or "control* trial" or "random* allocat*" or "difference in difference*" or difference-in-difference* or "double difference*" or "regression discontinuity" or "instrumental variable*" or "propensity score" or quasi-experiment* or "quasi experiment*" or QED or QES or matching or "IV estimation").ti,ab,kw. |
| 9 | ("systematic review*" or (systematic* adj2 review*) or meta-analy* or "meta analy*").ti,ab,kw. |
| 10 | or/7-9 |
| 11 | 3 and 6 and 10 |
| 12 | limit 11 to yr="2000 - 2019" |

**23. CAB Global Health:** <https://www.cabi.org/publishing-products/global-health/>

| **Results** | **Type** |
| --- | --- |
| 1 | or financ* or microfinance or guarantees or policies or policy or "international labour standard*" or "international labor standard*" or employ* or ALMPs or "active labour market*" or "active labor market*" or "value chain" or entrepreneur* or "social protection" or "social security" or (accountab* adj3 system*) or scholarship* or earn*).ti. |
| 2 | employment/ or employment opportunities/ or labour economics/ or labour market/ or inservice training/ or apprenticeship/ or on-the-job training/ or labour market/ or vocational training/ or job skills/ or entrepreneurship/ |
| 3 | or/1-2 |
| 4 | (Youth* or Teen* or "young people" or "young adult*" or "young person*" or adolescen* or "early adult*" or "aged from 15" or "aged under 35").ti. |
| 5 | adolescents/ or youth/ or young workers/ or rural youth/ or young adults/ |
| 6 | or/4-5 |
| 7 | (eval* or assess* or analy* or estimat* or effect or intervention* or measure*).ti,ab. |
| 8 | ("random* controlled trial" or "controlled clinical trial" or RCT or "control* trial" or "random* allocat*" or "difference in difference*" or difference-in-difference* or "double difference*" or "regression discontinuity" or "instrumental variable*" or "propensity score" or quasi-experiment* or "quasi experiment*" or QED or QES or matching or "IV estimation").ti,ab. |
| 9 | ("systematic review*" or (systematic* adj2 review*) or meta-analy* or "meta analy*").ti,ab. |
| 10 | or/7-10 |
| 11 | 3 and 6 and 11 |
| 12 | limit 12 to yr="2000 - 2019" |

**Annex 4: Reference List for definitions of Interventions and Outcomes**

1. Abebe, G. T., Caria, S., Fafchamps, M., Falco, P., Franklin, S., Quinn, S., & Shilpi, F. (2017). Job fairs: Matching firms and workers in a field experiment in Ethiopia. *World Bank Policy Research Working Paper*, (8092). Report: <http://www.simonrquinn.com/JobFairsExperiment.pdf>. Accessed 9 December 2019
2. Acevedo, P., Cruces, G., Gertler, P., & Martinez, S. (2017). *Living up to expectations: How job training made women better off and men worse off* (No. w23264). National Bureau of Economic Research. Report: <http://www.nber.org/papers/w23264>. Accessed 3 March 2020.
3. Ahmed, A., Chakravarty, S., Lundberg, M., & Nikolov, P. (2014). *The Role of Training Programs for Youth Employment in Nepal: Impact Evaluation Report on the Employment Fund.* World Bank. Report: <http://users.nber.org/~nikolovp/pubs/Neap_paper.pdf>. Accessed 28 February 2020.
4. Azevedo, T. A. D., Davis, J., & Charles, M. (2013). Testing what works in youth employment: Evaluating Kenya's Ninaweza program. *Baltimore, Maryland: International Youth Foundation*.
5. Beam, E. A. (2016). Do job fairs matter? Experimental evidence on the impact of job-fair attendance. *Journal of Development Economics*, *120*, 32-40.
6. Betcherman, G., Daysal, N. M., & Pagés, C. (2010). Do employment subsidies work? Evidence from regionally targeted subsidies in Turkey. *Labour Economics*, *17*(4), 710-722. <https://www.sciencedirect.com/science/article/abs/pii/S092753710900150X>
7. Blattman, C., Fiala, N., & Martinez, S. (2014). Generating skilled self-employment in developing countries: Experimental evidence from Uganda. *The Quarterly Journal of Economics*, *129*(2), 697-752.
8. Bordos, K., Csillag, M., & Scharl, A. (2015). *What works in wage subsidies for young people: A review of issues, theory, policies and evidence. ILO Working Papers, (994898973402676).* [*https://www.ilo.org/wcmsp5/groups/public/ed_emp/documents/publication/wcms_466538.pdf*](https://www.ilo.org/wcmsp5/groups/public/ed_emp/documents/publication/wcms_466538.pdf)*. Report: Accessed 19 December 2019.*
9. Business Dictionary. (2020). *Cost.* <http://www.businessdictionary.com/definition/cost.html>. Accessed 3 March 2020.
10. Business Directory. (2019). *Length of Service.* <http://www.businessdictionary.com/definition/length-of-service.html>. Accessed 20 December 2019.
11. CAPLA. (2019). *What is Prior Learning Assessment & Recognition (PLAR)/ Recognition of Prior Learning (RPL)?* <http://capla.ca/what-is-rpl/>. Accessed 19 December 2019.
12. Cellini, S. R., & Kee, J. E. (2010). Cost-effectiveness and cost-benefit analysis. *Handbook of practical program evaluation*, *3*. <https://onlinelibrary.wiley.com/doi/10.1002/9781119171386.ch24>. Accessed 10 December 2019.
13. Centre for Financial Inclusion. (2019). *Financial Inclusion Glossary*. <https://www.centerforfinancialinclusion.org/financial-inclusion-glossary>. Accessed 20 December 2019.
14. Chen, A. 2018, Grant, Investopedia. <https://www.investopedia.com/terms/g/grant.asp>, Accessed 28 February 2020.
15. Clarke, M., & Chalmers, I. (1998). Discussion sections in reports of controlled trials published in general medical journals: islands in search of continents?. *Jama*, *280*(3), 280-282.
16. EU. (2017). *EU Delegation to the Republic of Serbia: Public Works- a solution to unemployment.* <https://www.searchnewworld.com/search/search2.html?partid=rolbng&p=public+works+and+unemployment&subid=004>. Accessed 3 March 2020.
17. Eurofund. (2019). *Job Quality*. <https://www.eurofound.europa.eu/topic/job-quality>. Accessed 2o December 2019.
18. Farlex Financial Dictionary. (2012). *Job Creation*. <https://financial-dictionary.thefreedictionary.com/Job+Creation>. Accessed 20 December 2019.
19. Fiala, N. (2013). Stimulating microenterprise growth: Results from a loans, grants and training experiment in Uganda. *Grants and Training Experiment in Uganda (December 4, 2013)*.
20. Finch, C. (2018) *Difference Between Internship & Apprenticeship.* [https://work.chron.com/difference-between-internship-apprenticeship-29606.html. Accessed 20 December 2019.](https://work.chron.com/difference-between-internship-apprenticeship-29606.html.%20Accessed%2020%20December%202019.%20%20%20)
21. Flynn, R. (2013). *A Case Study of Rural Finance Self-Help Groups in Uganda and Their Impact on Poverty Alleviation and Development. Report:* [*https://digitalcollections.sit.edu/isp_collection/1688*](https://digitalcollections.sit.edu/isp_collection/1688)*. Accessed 20 December 2019.*
22. Forbes. (2019). *Small Business Ownership: Start Out Small With A Microfranchise*, <https://www.forbes.com/sites/allbusiness/2013/11/27/small-business-ownership-start-out-small-with-a-microfranchise/#65e6553e5a3c> . Accessed 20 December 2019.
23. Government of Netherlands. (2019). *Forms of crime*. <https://www.government.nl/topics/crime-and-crime-prevention/forms-of-crime> . Accessed 20 December 2019.
24. Granicus. (2019). *What Is Digital Government Service?* <https://granicus.com/dictionary/digital-government-services/>. Accessed 20 December 2019.
25. Heavey, D. (2017). *Examples of Successful Media Campaigns*. <https://thrivehive.com/examples-social-media-campaigns/>. Accessed 28 February 2020.
26. Hirshleifer, S., McKenzie, D., Almeida, R., & Ridao‐Cano, C. (2016). The impact of vocational training for the unemployed: experimental evidence from Turkey. *The Economic Journal*, *126*(597), 2115-2146.
27. HR. (2019). *The Differences between Reskilling and Upskilling. Blog*. <https://www.hrinasia.com/general/the-differences-between-reskilling-and-upskilling/>. Accessed 20 December 2019.
28. Ibarraran, P., Ripani, L., Taboada, B., Villa, J. M., & Garcia, B. (2014). Life skills, employability and training for disadvantaged youth: Evidence from a randomized evaluation design. *IZA Journal of Labor & Development*, *3*(1), 1-24.
29. International Labour Organization. (2010). *A skilled workforce for strong, sustainable and balanced growth: a G20 training strategy. Report:* <https://www.ilo.org/skills/pubs/WCMS_151966/lang--en/index.htm>. Accessed 20 December 2019.
30. International Labour Organization. (2017). *World social protection report 2017–19: Universal social protection to achieve the Sustainable Development Goals. Report:* [*https://www.ilo.org/global/publications/books/WCMS_604882/lang--en/index.htm*](https://www.ilo.org/global/publications/books/WCMS_604882/lang--en/index.htm)*. Accessed 28 February 2020.*
31. International Labour Organization. (2019). *Conventions and Recommendations*. <https://www.ilo.org/global/standards/introduction-to-international-labour-standards/conventions-and-recommendations/lang--en/index.htm>. Accesed19 December 2019.
32. International Labour Organization. (2020). *Social Protection.* <https://www.ilo.org/global/topics/social-security/lang--en/index.htm>. Accessed 28 February 2010.
33. International Labour Organization. (n.d.). Employment Research Brief: Employment Policy Implementation Mechanisms across countries. <https://www.ilo.org/wcmsp5/groups/public/---ed_emp/documents/publication/wcms_613372.pdf>. Accessed 3 March 2020.
34. Innovaccess - Intellectual Property Portal. (2019). *Business creation.* <http://www.innovaccess.eu/business-creation>. Accessed 20 December 2019.
35. International Labour Office. Skills and Employability Department (EMP/SKILLS). (2012). *Upgrading informal apprenticeship: a resource guide for Africa*. International Labour Office, Geneva, Switzerland. Report: <https://www.ilo.org/skills/pubs/WCMS_171393/lang--en/index.htm>. Accessed 19 December 2019.
36. Investopedia. (2019a). *Crowdfunding*. <https://www.investopedia.com/terms/c/crowdfunding.asp>. Accessed 20 December 2019.
37. Investopedia. (2019b). *Microfinance*. <https://www.investopedia.com/terms/m/microfinance.asp>. Accessed 20 December 2019.
38. Invetopedia 2019, Social Impact Bond (SIB). <https://www.investopedia.com/terms/s/social-impact-bond.asp>. Accessed 20 December 2019.
39. Kagan, J.(2019). *Guaranteed Loan – Definition, Inestopedia.* <https://www.investopedia.com/terms/g/guaranteed-loan.asp>. Accessed 28 February 2020.
40. Kluve, J., Puerto, S., Stoeterau, J., Weidenkaff, F., Witte, M., Robalino, D., ... & Rother, F. (2014). Protocol: Interventions to improve labour market outcomes of youth: A systematic review of active labour market programmes. *Campbell Systematic Reviews*, *10*(1), 1-109. Protocol: <https://onlinelibrary.wiley.com/doi/full/10.1002/CL2.12320>. Accessed 10 December 2019.
41. Lewin, K. M. Educational access, equity, and development: planning to make rights realities. <https://unesdoc.unesco.org/ark:/48223/pf0000235003>. Accessed 10 December 2019.
42. Lippman, L. H., Ryberg, R., Carney, R., & Moore, K. A. (2015). Workforce Connections: Key “soft skills” that foster youth workforce success: toward a consensus across fields. *Washington, DC: Child Trends*. <https://www.childtrends.org/wp-content/uploads/2015/06/2015-24WFCSoftSkills1.pdf>. Accessed 10 February 2020.
43. Longman. (2019). *Longman Business Dictionary*. <https://www.ldoceonline.com/dictionary/job-placement>. Accessed 20 December 2019.
44. McGuire, G. (2002). *Trade in services: market access opportunities and the benefits of liberalization for developing economies* (Vol. 19). Univ of California Press.
45. Mind Tools. (2019). *What Is Personal Empowerment?* <https://www.mindtools.com/pages/article/personal-empowerment.htm>. Accessed 20 December 2019.
46. Nutz, N., & Sievers, M. (2015). *A rough guide to value chain development: How to create employment and improve working conditions in targeted sectors*. ILO. *Report:* [*https://www.ilo.org/wcmsp5/groups/public/---ed_emp/---emp_ent/---ifp_seed/documents/publication/wcms_366005.pdf*](https://www.ilo.org/wcmsp5/groups/public/---ed_emp/---emp_ent/---ifp_seed/documents/publication/wcms_366005.pdf)*. Accessed 20 December 2019.*
47. Organization for Economic Co-operation and Development (OECD). (2012). *Transferable Skills Training for Researchers: Supporting Career Development and Research*. OECD Publishing. <https://read.oecd-ilibrary.org/science-and-technology/transferable-skills-training-for-researchers_9789264179721-en#page4>. Accessed 20 December 2019.
48. Ponizovskiy, V., Grigoryan, L., Kühnen, U., & Boehnke, K. (2019). Social construction of the value–behavior relation. *Frontiers in psychology*, *10*, 934. <https://doi.org/10.3389/fpsyg.2019.00934>. Accessed 20 December 2019.
49. Program Success Center for Sexual & Reproductive Health. (2019). *Training of Trainers (TOT).* <https://www.etr.org/ebi/training-ta/types-of-services/training-of-trainers/>. Accessed 19 December 2019.
50. Scism, T. E. (1974). Employee Mobility in the Federal Service: A Description of Some Recent Data. *Public Administration Review*, *34*(3), 247-254
51. Small Business Development Corporation. (2019). *Essential business skills*. <https://www.smallbusiness.wa.gov.au/business-advice/starting-your-business/business-skills>. Accessed 19 December 2019.
52. UNESCO. (2012). *International Standard Classification of Education ISCED 2011*. Montreal. [https://www.google.com/url?sa=t&rct=j&q=&esrc=s&source=web&cd=4&cad=rja&uact=8&ved=2ahUKEwj176K-h8LmAhVuD2MBHf8mAcIQFjADegQIARAC&url=http%3A%2F%2Fuis.unesco.org%2Fsites%2Fdefault%2Ffiles%2Fdocuments%2Finternational-standard-classification-of-education-isced-2011-en.pdf&usg=AOvVaw2BYYJ4Vx3zdUbGuqIGbNCD](https://www.google.com/url?sa=t&rct=j&q=&esrc=s&source=web&cd=4&cad=rja&uact=8&ved=2ahUKEwj176K-h8LmAhVuD2MBHf8mAcIQFjADegQIARAC&url=http%3A%2F%2Fuis.unesco.org%2Fsites%2Fdefault%2Ffiles%2Fdocuments%2Finternational-standard-classification-of-education-isce). Accessed 19 December 2019.
53. UNESCO. (2019*). Glossary****:*** *Non-formal education*. <http://uis.unesco.org/en/glossary-term/non-formal-education>. Accessed 19 December 2019.
54. UNESCO. (2019a). *TVETipedia Glossary.* <https://unevoc.unesco.org/go.php?q=TVETipedia+Glossary+A-Z&term=Technical+and+vocational+education+and+training>. Acccessed 19 December 2019.
55. UNESCO. (2019b). *TVETipedia Glossary, On-the-job training (OJT)*. <https://unevoc.unesco.org/go.php?q=TVETipedia+Glossary+A-Z&filt=&id=346>. Accessed 19 December 2019.
56. UNESCO. (2019c). *Glossary: Completion rate.* <http://uis.unesco.org/en/glossary-term/completion-rate>. Accessed 20 December 2019.
57. UNESCO. (2019d). *TVETipedia Glossary.* <https://unevoc.unesco.org/go.php?q=TVETipedia+Glossary+A-Z&term=Technical+and+vocational+education+and+training>. Accessed 20 December 2019.
58. UNESCO. (2019e). *Digital skills critical for jobs and social inclusion.* <https://en.unesco.org/news/digital-skills-critical-jobs-and-social-inclusion>. Accessed 20 December 2019.
59. USA Legal. (2019). *Employment Status Law and Legal Definition*. <https://definitions.uslegal.com/e/employment-status/>. Accessed 20 December 2019.
60. USA. Department of Education. (2019*). Career Guidance and Counseling Programs.* <https://www2.ed.gov/about/offices/list/ovae/pi/cte/cgcp.html>. Accessed 20 December 2019.
61. USA. State of California Department of Industrial Relations. (2020). *Public Works*. <https://www.dir.ca.gov/Public-Works/PublicWorks.html>. Accessed 28 February 2020.
62. Vlăsceanu, L., Grünberg, L., & Pârlea, D. (2004). *Quality assurance and accreditation: A glossary of basic terms and definitions* (p. 25). Bucharest: Unesco-Cepes. https://www.aracis.ro/wp-content/uploads/2019/08/Glossary_07_05_2007.pd
63. Vvob. (2019). *Our vision on quality education*. <https://www.vvob.org/en/education/our-vision-on-quality-education>. Accessed 20 December 2019.
64. World Bank. (2020). *Small and Medium Enterprises (SMEs) Finance.* <https://www.worldbank.org/en/topic/smefinance>. Accessed 28 February 2020.

1. CAPLA = Canadian Association for Prior Learning Assessment [↑](#footnote-ref-1)
